# Author Correction: Mechanical properties of rubble pile asteroids (Dimorphos, Itokawa, Ryugu, and Bennu) through surface boulder morphological analysis

**DOI:** 10.1038/s41467-024-54679-z

**Published:** 2024-12-12

**Authors:** Colas Q. Robin, Alexia Duchene, Naomi Murdoch, Jean-Baptiste Vincent, Alice Lucchetti, Maurizio Pajola, Carolyn M. Ernst, R. Terik Daly, Olivier S. Barnouin, Sabina D. Raducan, Patrick Michel, Masatochi Hirabayashi, Alexander Stott, Gabriela Cuervo, Erica R. Jawin, Josep M. Trigo-Rodriguez, Laura M. Parro, Cecily Sunday, Damien Vivet, David Mimoun, Andrew S. Rivkin, Nancy L. Chabot

**Affiliations:** 1 Institut Supérieur de l’Aéronautique et de l’Espace (ISAE-SUPAERO), Université de Toulouse, Toulouse, France; 2 DLR Institute of Planetary Research, Berlin, Germany; 3 INAF-Astronomical Observatory of Padova, Padova, Italy; 4 Johns Hopkins University Applied Physics Laboratory, Laurel, MD USA; 5 University of Bern, Bern, Switzerland; 6 Côte d’Azur University, Côte d’Azur Observatory, CNRS, Lagrange Laboratory, Nice, France; 7 The University of Tokyo, Department of Systems Innovation, School of Engineering, Tokyo, Japan; 8 Georgia Institute of Technology, Atlanta, GA 30332 USA; 9 Smithonian National Air and Space Museum, Washington, DC USA; 10 Institute of Space Sciences (CSIC-IEEC), Campus UAB, Carrer Can Magrans s/n, Cerdanyola del Valles, Barcelona, Catalonia Spain; 11 IUFACyT, Alicante University, San Vicente del Raspeig, 03080 Alicante, Spain; 12 University of Maryland, College Park, MD USA

**Keywords:** Asteroids, comets and Kuiper belt, Asteroids, comets and Kuiper belt, Geomorphology

Correction to: *Nature Communications* 10.1038/s41467-024-50147-w, published online 30 July 2024

In this article the funding from the Spanish project PID2021-128062NB-I00 funded by MCIN/AEI was omitted. The original article has been corrected.

