# Supplementary material for: Mechanical properties of rubble pile asteroids (Dimorphos, Itokawa, Ryugu, and Bennu) through surface boulder morphological analysis
Source: Nat Commun. 2024 Jul 30;15:6203. doi: 10.1038/s41467-024-50147-w (PMC11289397; doi:10.1038/s41467-024-50147-w)
Supplement: Supplementary file 1 — Supplementary Information [file 41467_2024_50147_MOESM1_ESM.pdf]

Supplementary Information

Mechanical properties of rubble pile asteroids (Dimorphos, Itokawa, Ryugu, and Bennu) through surface boulder morphological analysis

Colas Q. Robin[1], Alexia Duchene[1], Naomi Murdoch[1], Jean-Baptiste Vincent[2], Alice Lucchetti[3], Maurizio Pajola[3], Carolyn M. Ernst[4], R. Terik Daly[4], Olivier S. Barnouin[4], Sabina D. Raducan[5], Patrick Michel[6,7], Masatochi Hirabayashi[8], Alexander Stott[1], Gabriela Cuervo[1], Erica R. Jawin[9], Josep M. Trigo-Rodriguez[10], Laura M. Parro[11], Cecily Sunday[1,12], Damien Vivet[1], David Mimoun[1], Andrew S. Rivkin[4], and Nancy L. Chabot[4]

[1]Institut Supérieur de l'Aéronautique et de l'Espace (ISAE-SUPAERO), Université de Toulouse, Toulouse, France, colas.robin@isae.fr
[2]DLR Institute of Planetary Research, Berlin, Germany
[3]INAF-Astronomical Observatory of Padova, Padova, Italy
[4]Johns Hopkins University Applied Physics Laboratory, Laurel, MD, USA
[5]University of Bern, Bern, Switzerland
[6]Côte d'Azur University,Côte d'Azur Observatory, CNRS, Lagrange Laboratory, Nice, France
[7]The University of Tokyo, Department of Systems Innovation, School of Engineering, Tokyo, Japan
[8]Georgia Institute of Technology, Atlanta, GA, 30332, USA
[9]Smithonian National Air and Space Museum, Washington, DC, USA
[10]Institute of Space Sciences (CSIC-IEEC), Campus UAB, Carrer Can Magrans s/n, Cerdanyola del Valles, Barcelona, Catalonia, Spain
[11]IUFACyT, Alicante University, San Vicente del Raspeig, 03080, Alicante, Spain
12University of Maryland, USA

Supplementary Figures

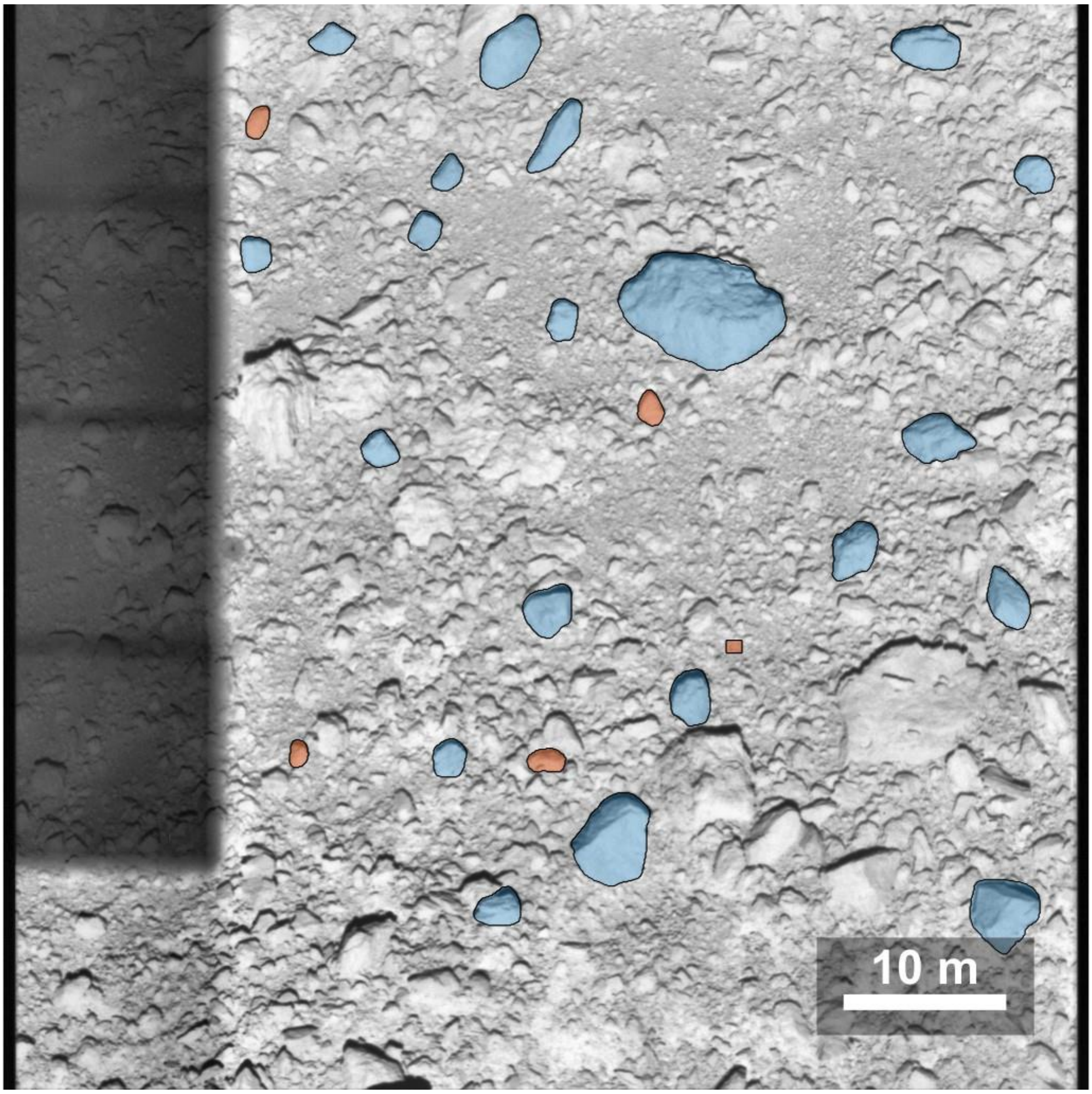

Supplementary Figure 1 | Itokawa surface. Asteroid (25143) Itokawa taken by AMICA (st_2530297837_v). The image contrast has been enhanced with a CLAHE filtering for visualisation purposes only. Boulders selected and analysed are coloured in blue and red. The red boulders indicate the smaller resolved boulders (<30 px), which haven't been included in the analysis of the resolution dependant morphological parameters.

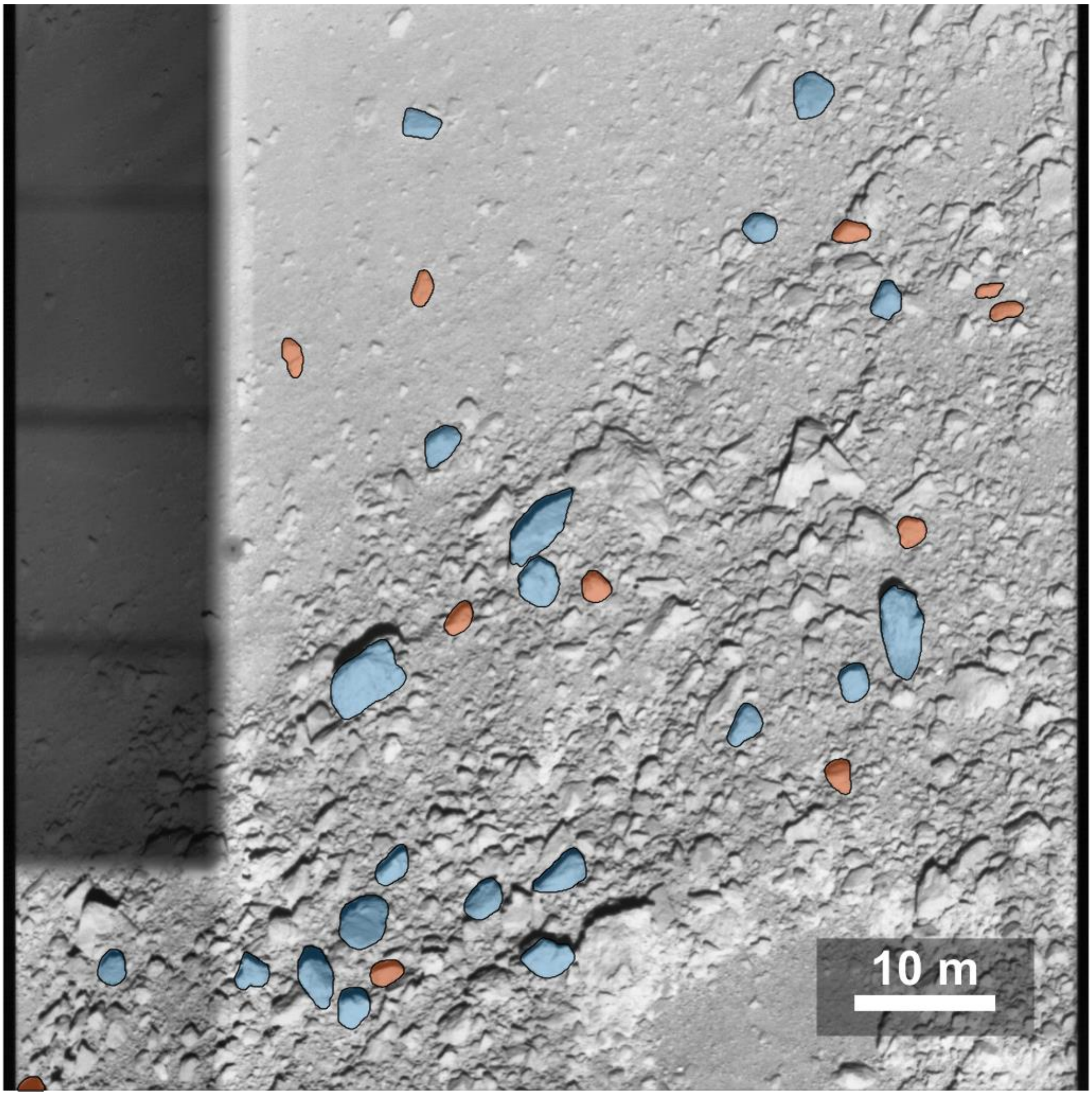

Supplementary Figure 2 | Itokawa surface. Asteroid (25143) Itokawa taken by AMICA (st_2532629277_v). The image contrast has been enhanced with a CLAHE filtering for visualisation purposes only. Boulders selected and analysed are coloured in blue and red. The red boulders indicate the smaller resolved boulders (<30 px), which haven't been included in the analysis of the resolution dependant morphological parameters.

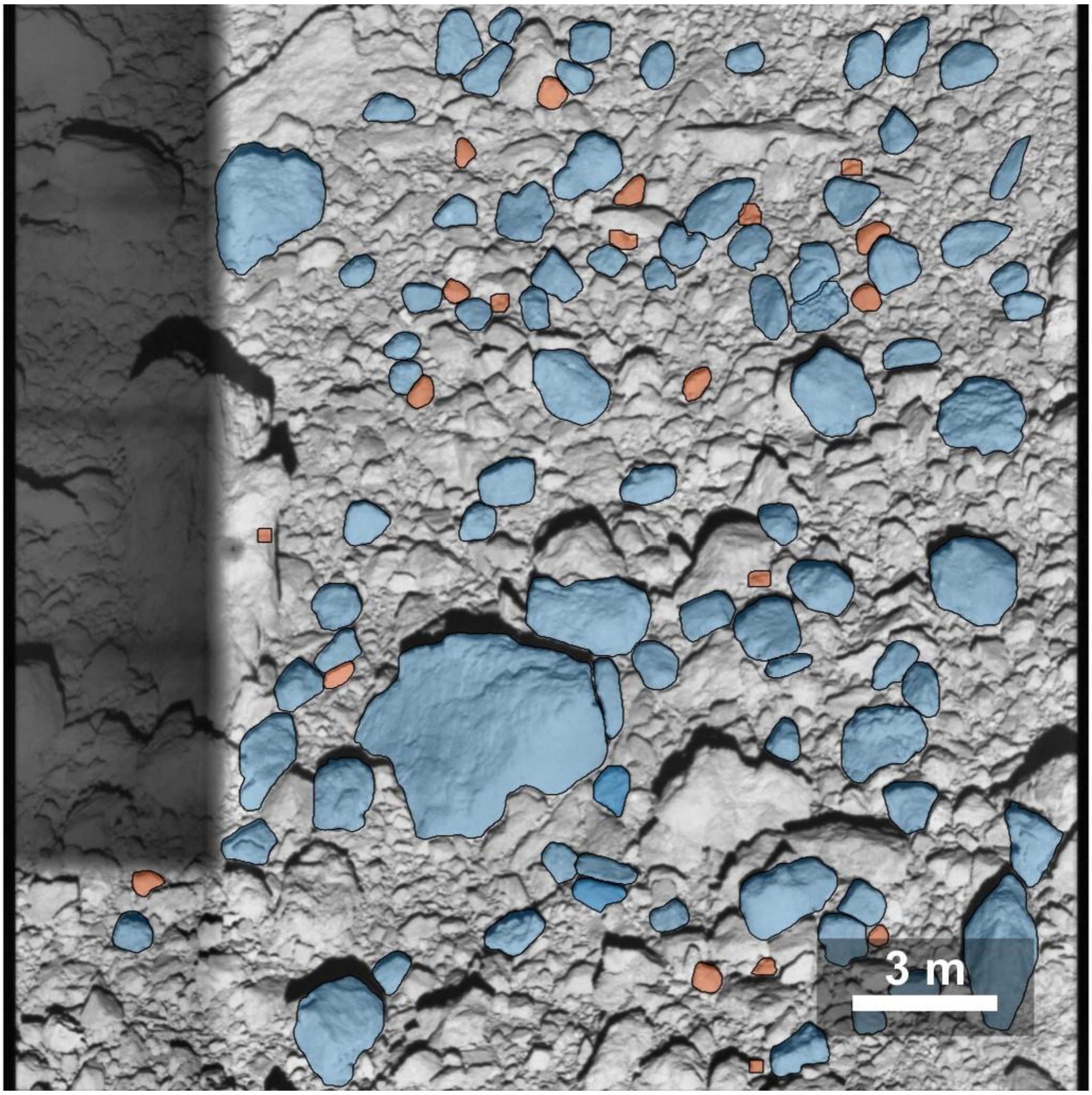

Supplementary Figure 3 | Itokawa surface. Asteroid (25143) Itokawa taken by AMICA (st_2539429953_v). The image contrast has been enhanced with a CLAHE filtering for visualisation purposes only. Boulders selected and analysed are coloured in blue and red. The red boulders indicate the smaller resolved boulders (<30 px), which haven't been included in the analysis of the resolution dependant morphological parameters.

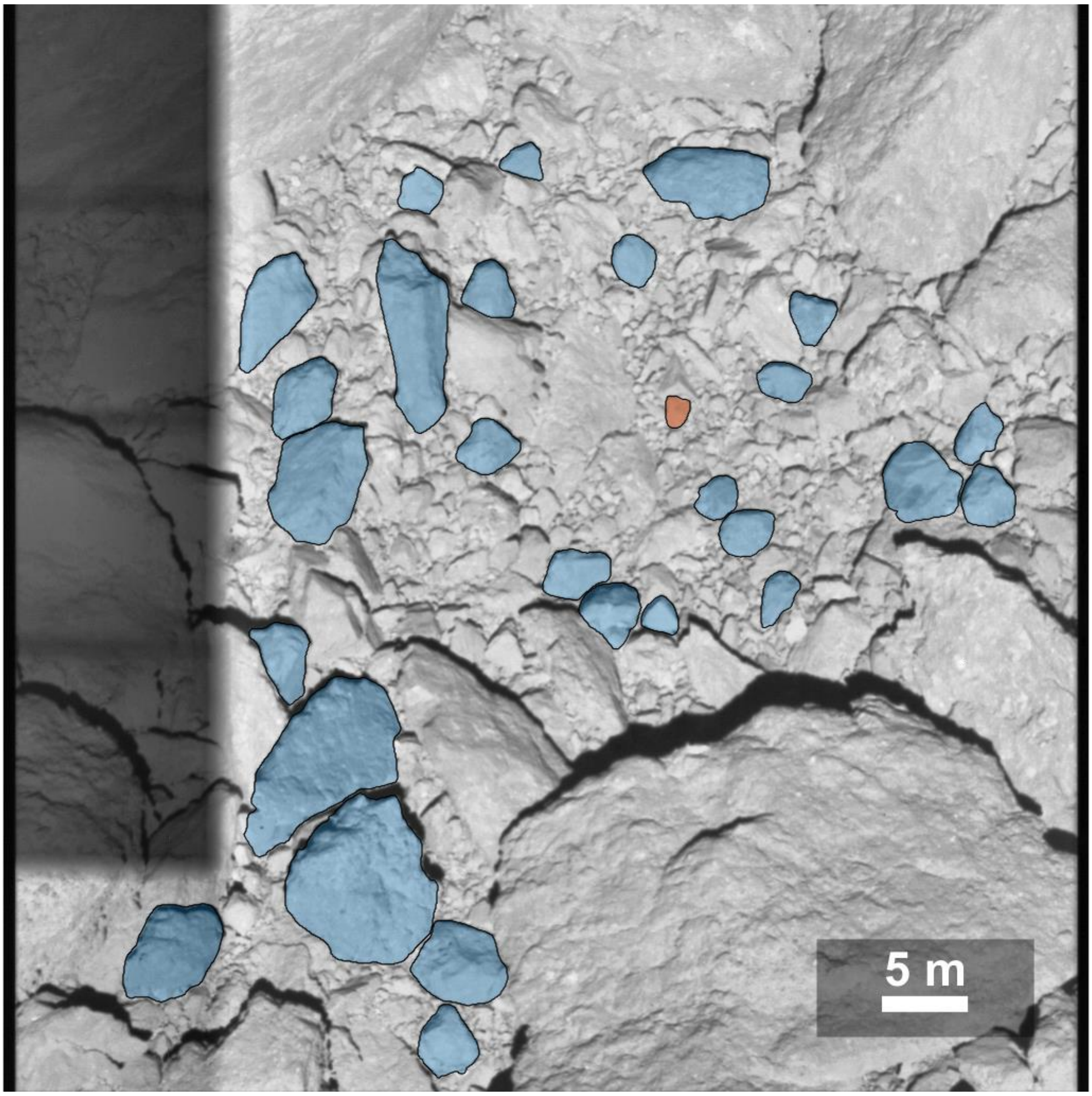

Supplementary Figure 4 | Itokawa surface. Asteroid (25143) Itokawa taken by AMICA (st_2539444467_v). The image contrast has been enhanced with a CLAHE filtering for visualisation purposes only. Boulders selected and analysed are coloured in blue and red. The red boulders indicate the smaller resolved boulders (<30 px), which haven't been included in the analysis of the resolution dependant morphological parameters.

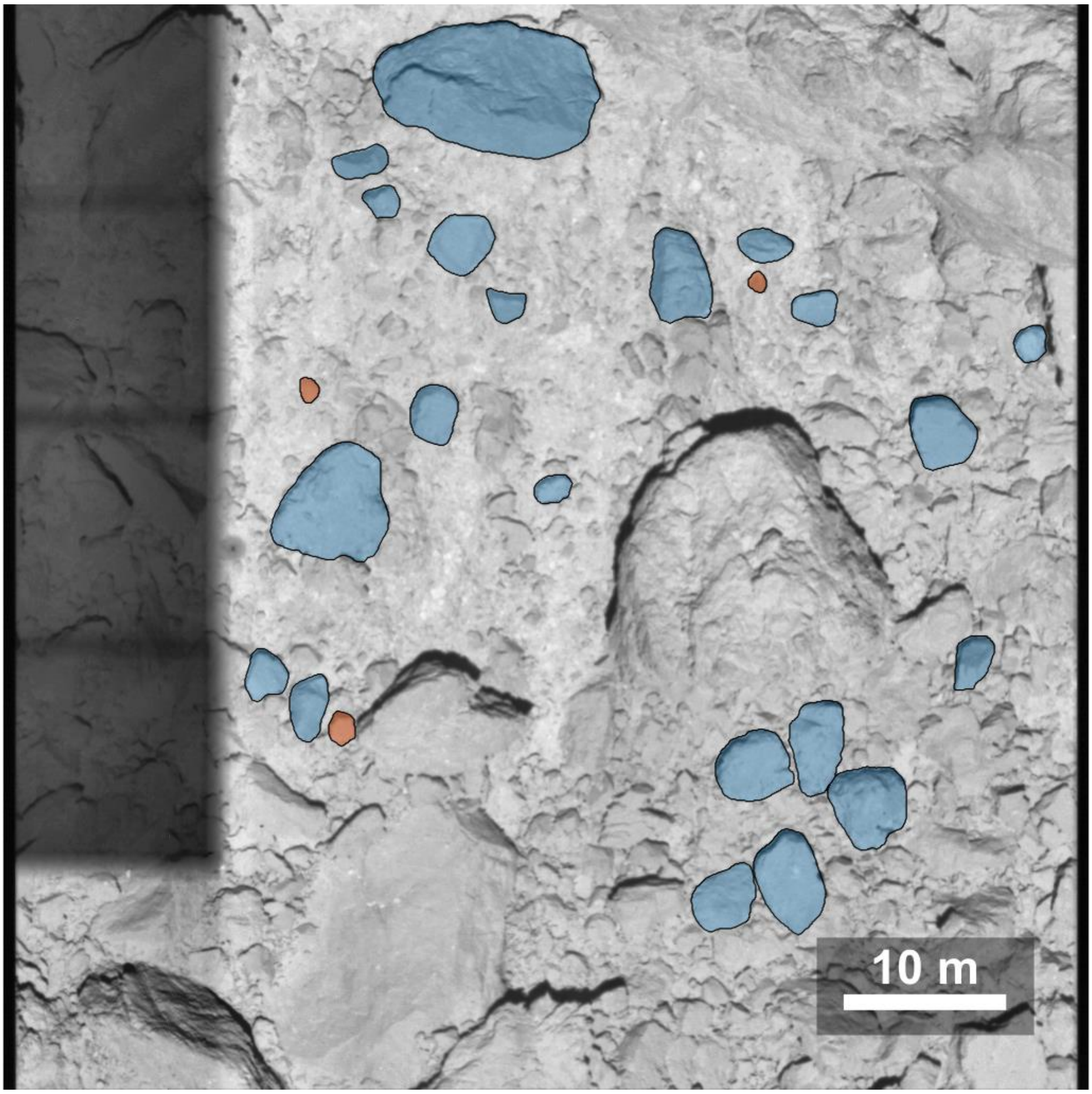

Supplementary Figure 5 | Itokawa surface. Asteroid (25143) Itokawa taken by AMICA (st_2539451609_v). The image contrast has been enhanced with a CLAHE filtering for visualisation purposes only. Boulders selected and analysed are coloured in blue and red. The red boulders indicate the smaller resolved boulders (<30 px), which haven't been included in the analysis of the resolution dependant morphological parameters.

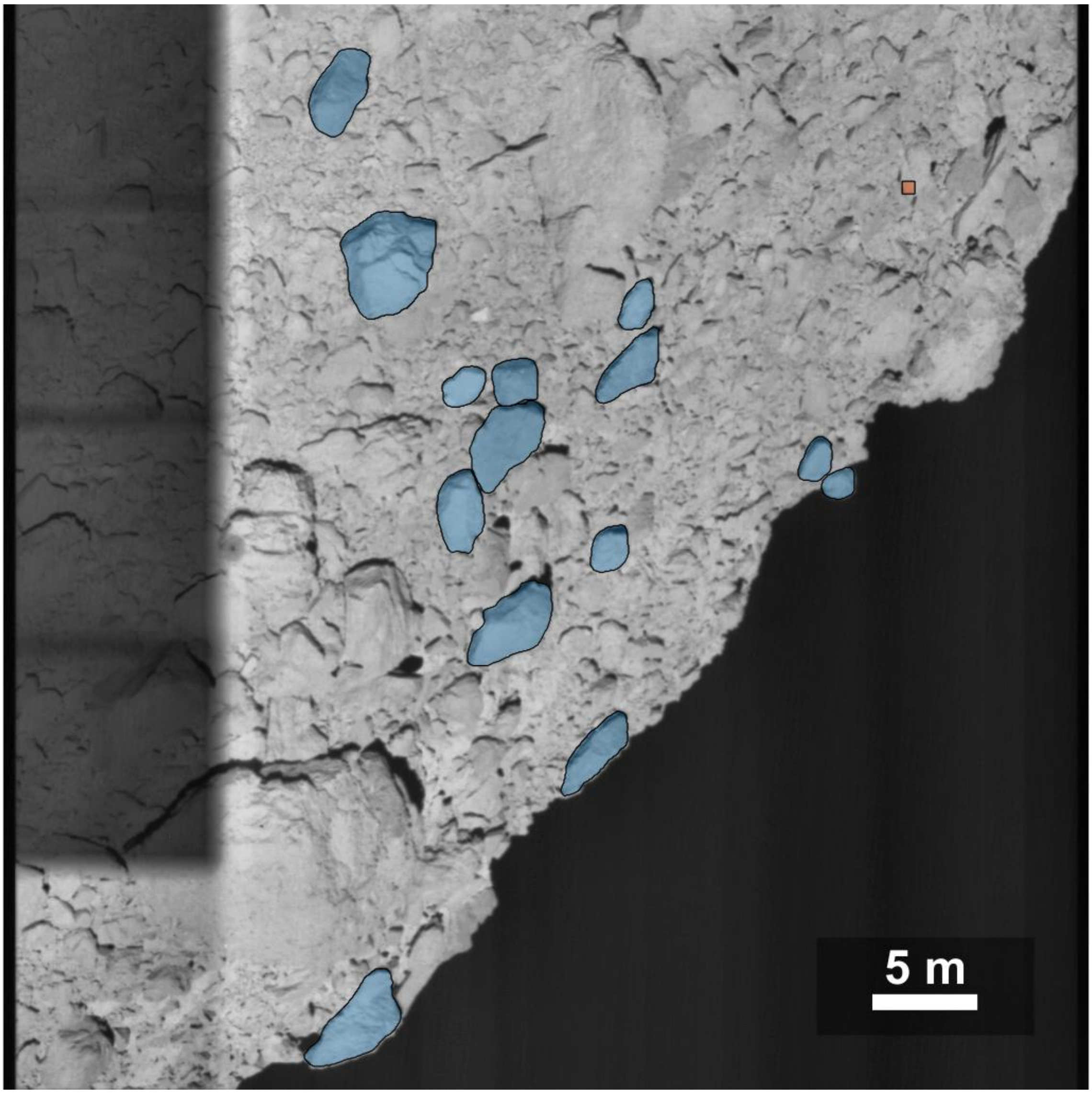

Supplementary Figure 6 | Itokawa surface. Asteroid (25143) Itokawa taken by AMICA (st_2539467169_v). The image contrast has been enhanced with a CLAHE filtering for visualisation purposes only. Boulders selected and analysed are coloured in blue and red. The red boulders indicate the smaller resolved boulders (<30 px), which haven't been included in the analysis of the resolution dependant morphological parameters.

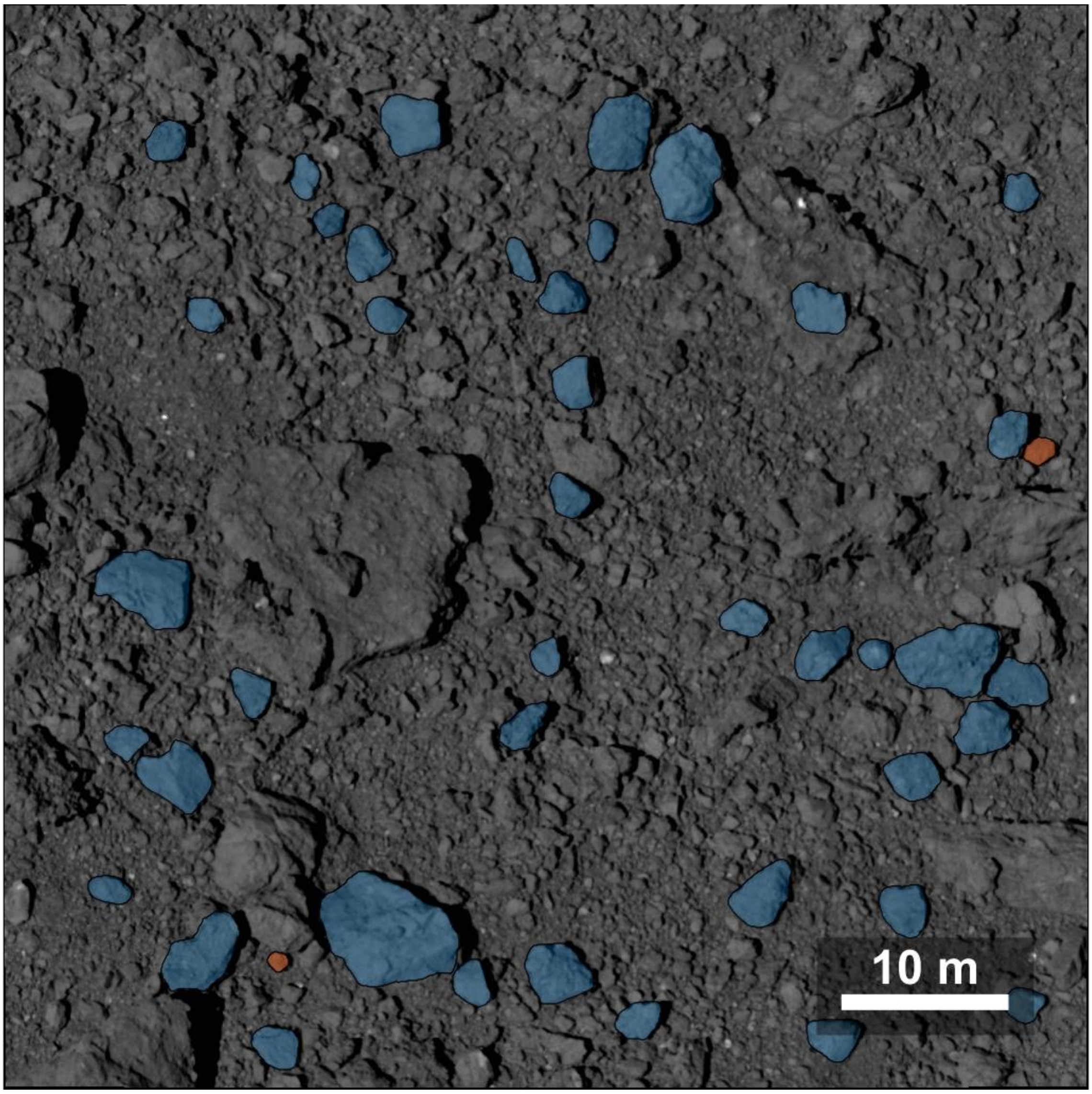

Supplementary Figure 7 | Ryugu surface. Asteroid (162173) Ryugu taken by ONC-T (hyb2_onc_20180921_034938_tvf_l2c). The image contrast has been enhanced with a CLAHE filtering for visualisation purposes only. Boulders selected and analysed are coloured in blue and red. The red boulders indicate the smaller resolved boulders (<30 px), which haven't been included in the analysis of the resolution dependant morphological parameters.

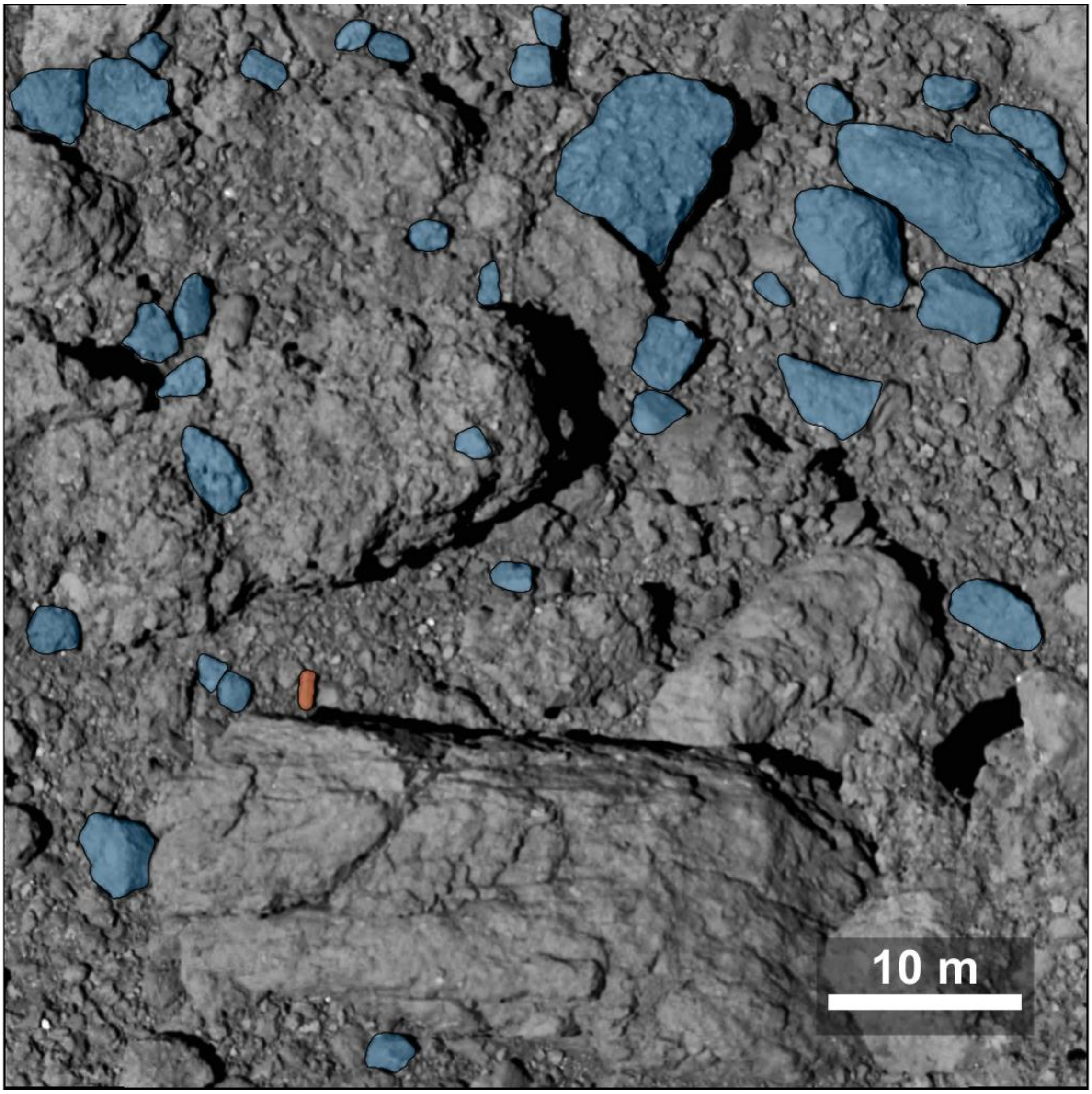

Supplementary Figure 8 | Ryugu surface. Asteroid (162173) Ryugu taken by ONC-T (hyb2_onc_20180921_040634_tvf_l2c). The image contrast has been enhanced with a CLAHE filtering for visualisation purposes only. Boulders selected and analysed are coloured in blue and red. The red boulders indicate the smaller resolved boulders (<30 px), which haven't been included in the analysis of the resolution dependant morphological parameters.

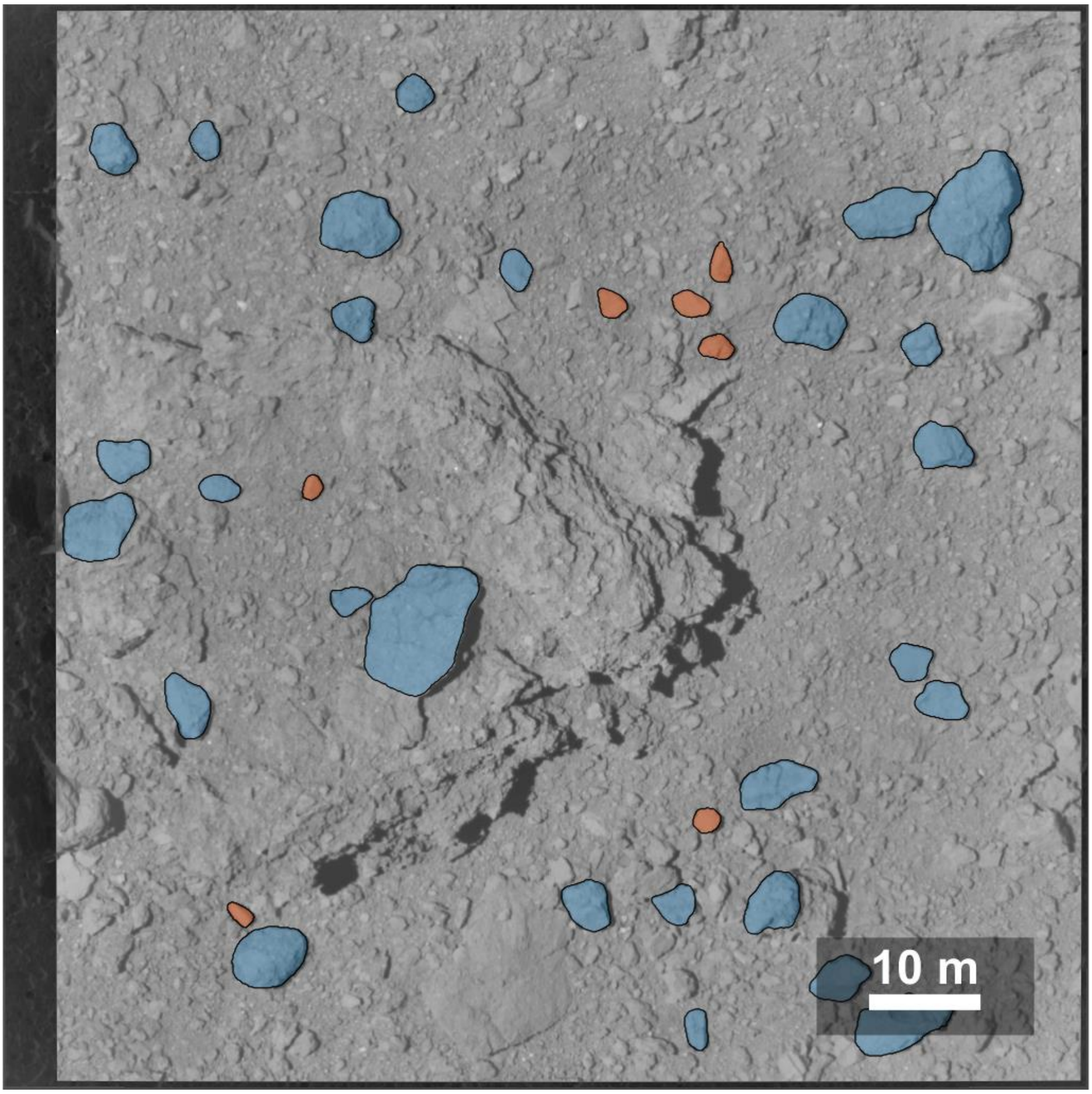

Supplementary Figure 9 | Ryugu surface. Asteroid (162173) Ryugu taken by ONC-T (hyb2_onc_20181003_003121_tnf_l2c). The image contrast has been enhanced with a CLAHE filtering for visualisation purposes only. Boulders selected and analysed are coloured in blue and red. The red boulders indicate the smaller resolved boulders (<30 px), which haven't been included in the analysis of the resolution dependant morphological parameters.

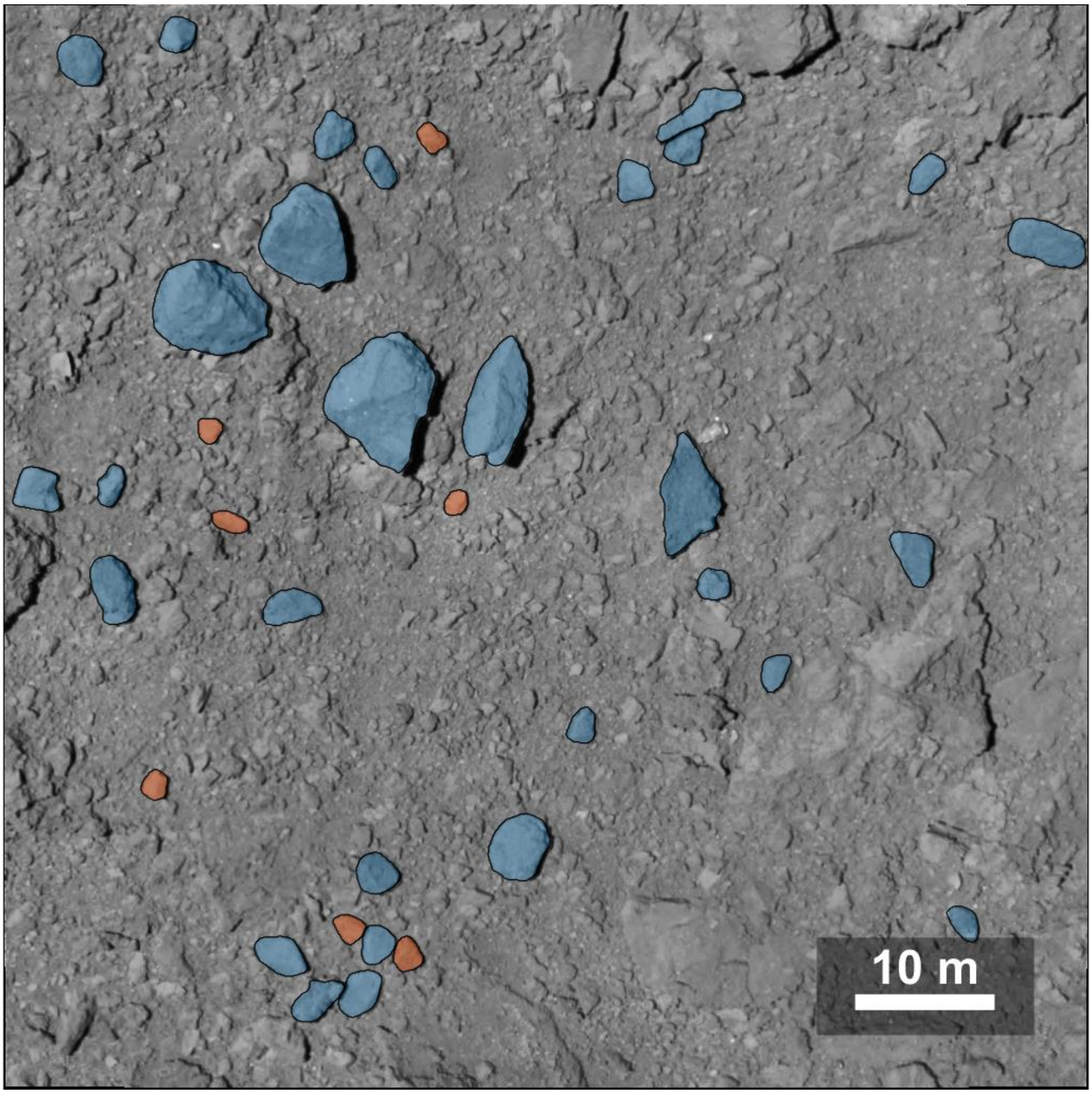

Supplementary Figure 10 | Ryugu surface. Asteroid (162173) Ryugu taken by ONC-T (hyb2_onc_20181003_021156_tvf_l2c). The image contrast has been enhanced with a CLAHE filtering for visualisation purposes only. Boulders selected and analysed are coloured in blue and red. The red boulders indicate the smaller resolved boulders (<30 px), which haven't been included in the analysis of the resolution dependant morphological parameters.

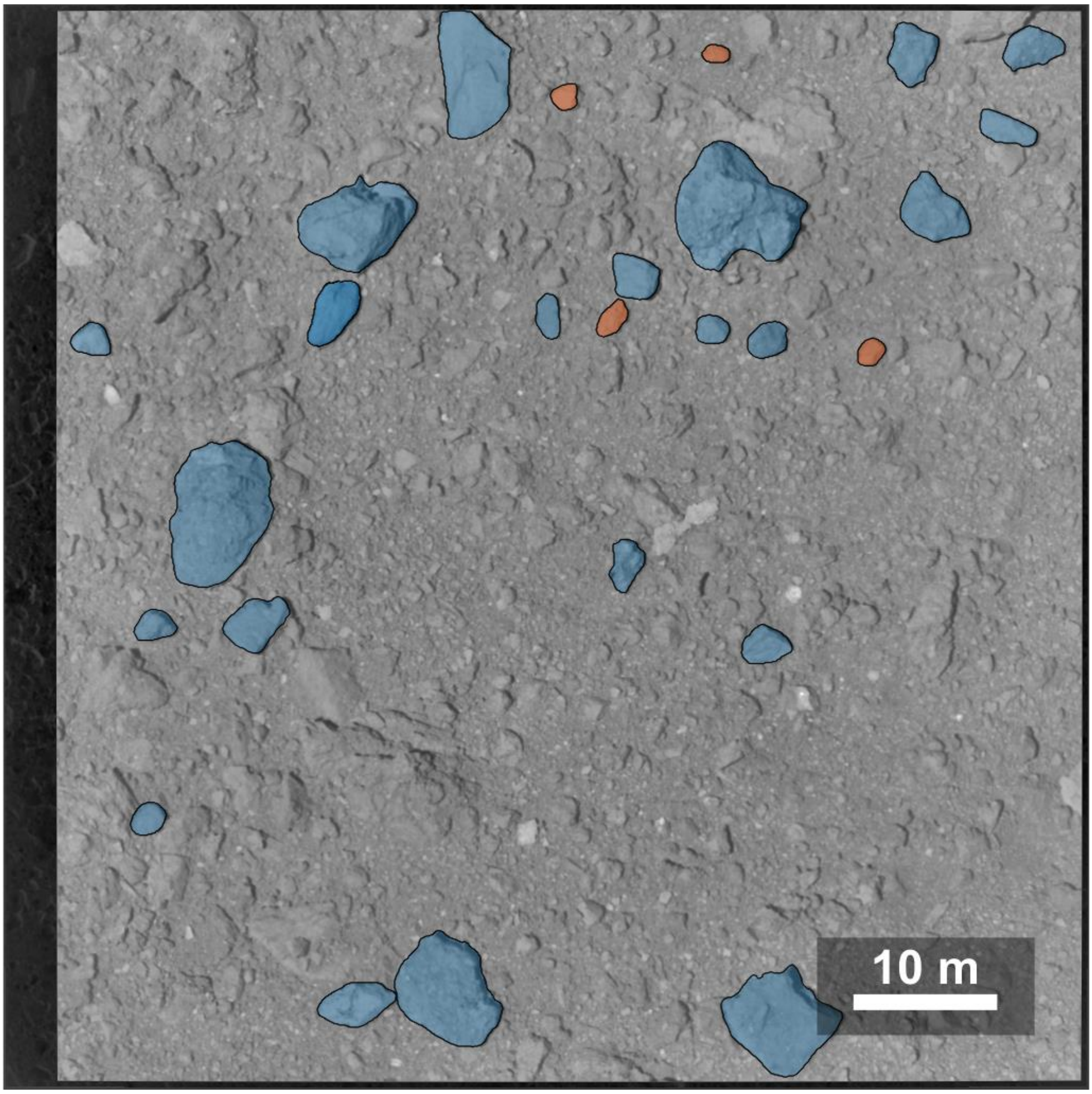

Supplementary Figure 11 | Ryugu surface. Asteroid (162173) Ryugu taken by ONC-T (hyb2_onc_20181015_130841_tuf_l2c). The image contrast has been enhanced with a CLAHE filtering for visualisation purposes only. Boulders selected and analysed are coloured in blue and red. The red boulders indicate the smaller resolved boulders (<30 px), which haven't been included in the analysis of the resolution dependant morphological parameters.

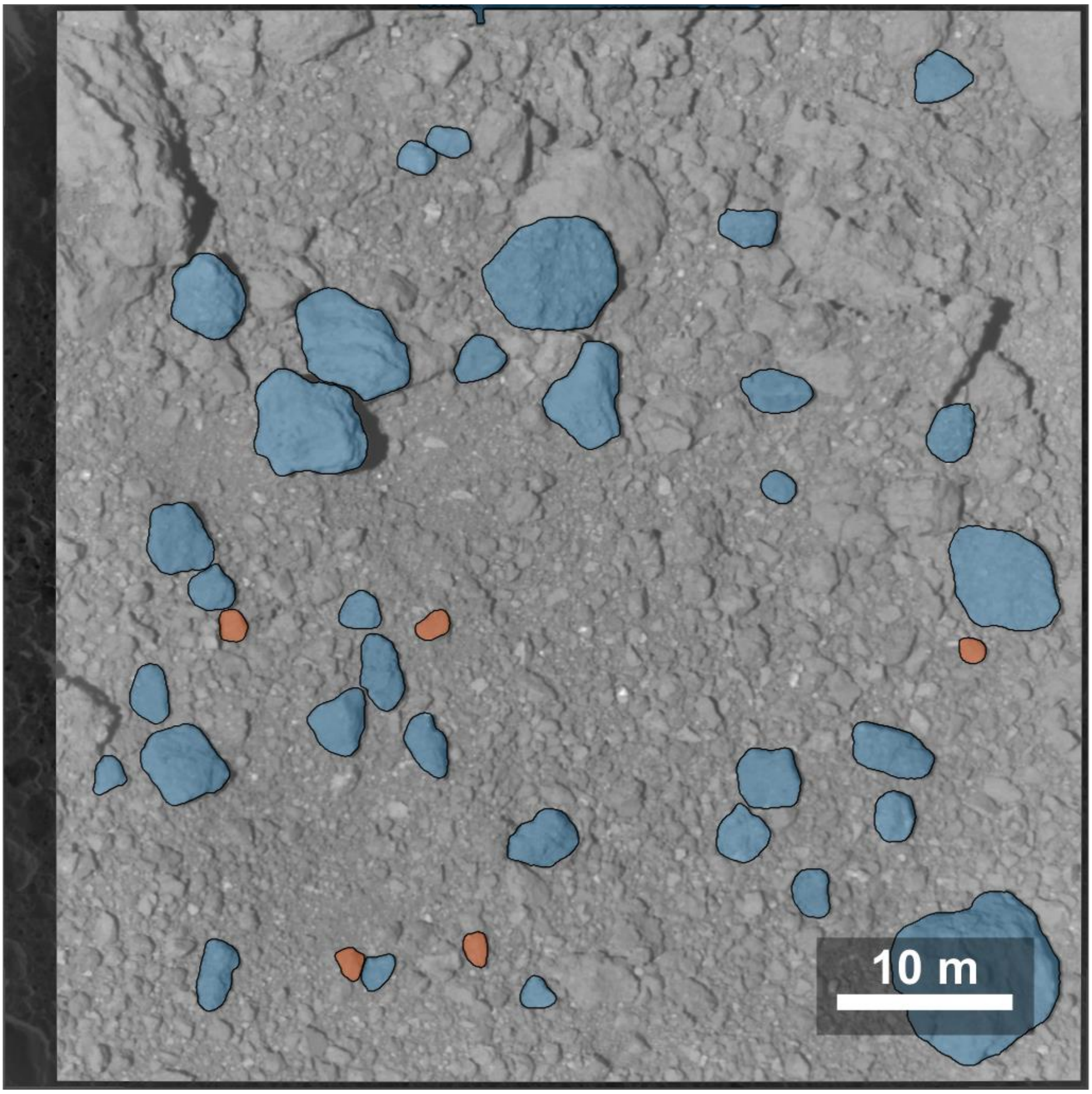

Supplementary Figure 12 | Ryugu surface. Asteroid (162173) Ryugu taken by ONC-T (hyb2_onc_20181015_133137_tpf_l2c). The image contrast has been enhanced with a CLAHE filtering for visualisation purposes only. Boulders selected and analysed are coloured in blue and red. The red boulders indicate the smaller resolved boulders (<30 px), which haven't been included in the analysis of the resolution dependant morphological parameters.

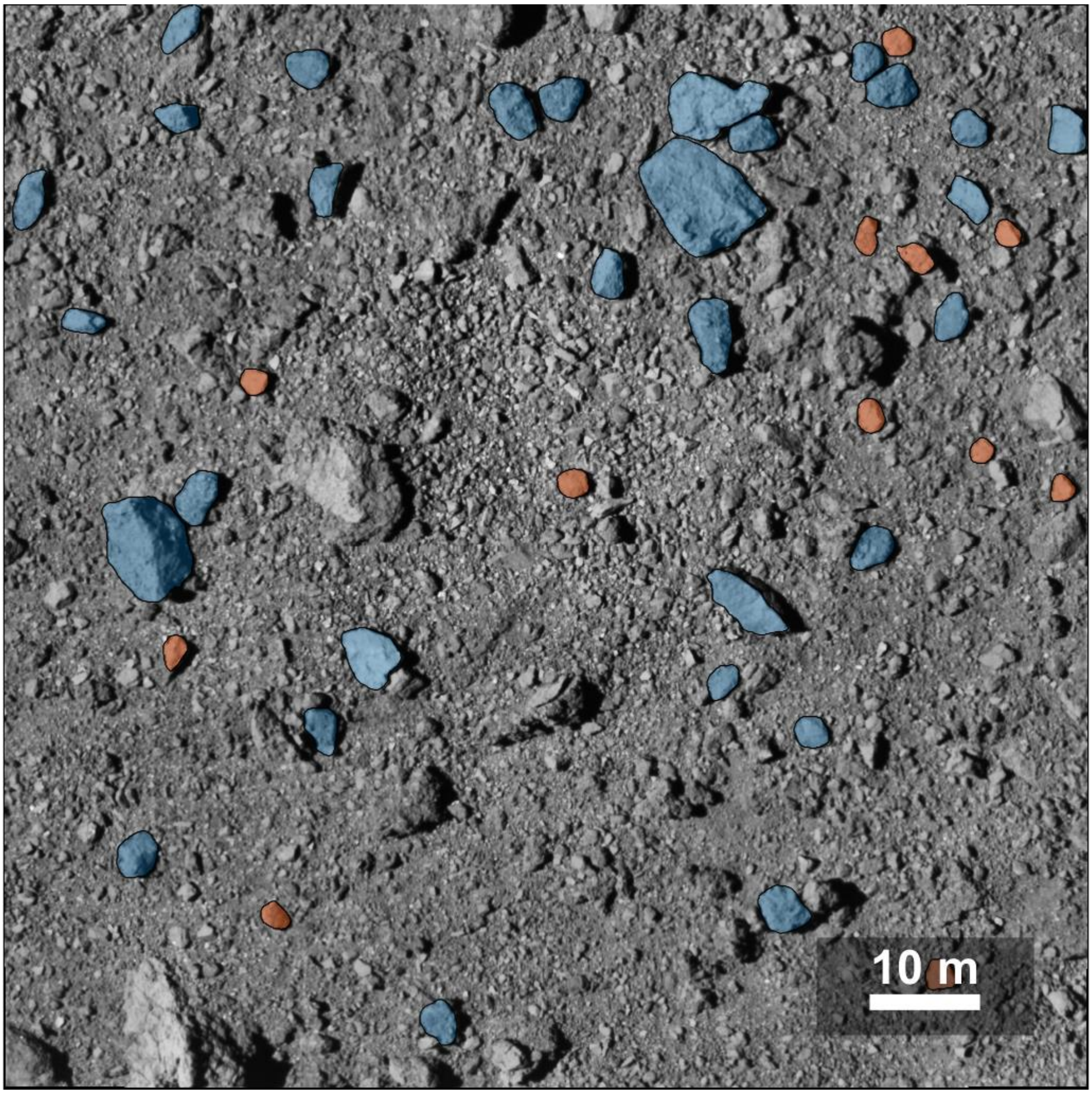

Supplementary Figure 13 | Ryugu surface. Asteroid (162173) Ryugu taken by ONC-T (hyb2_onc_20190710_232532_tvf_l2c). The image contrast has been enhanced with a CLAHE filtering for visualisation purposes only. Boulders selected and analysed are coloured in blue and red. The red boulders indicate the smaller resolved boulders (<30 px), which haven't been included in the analysis of the resolution dependant morphological parameters.

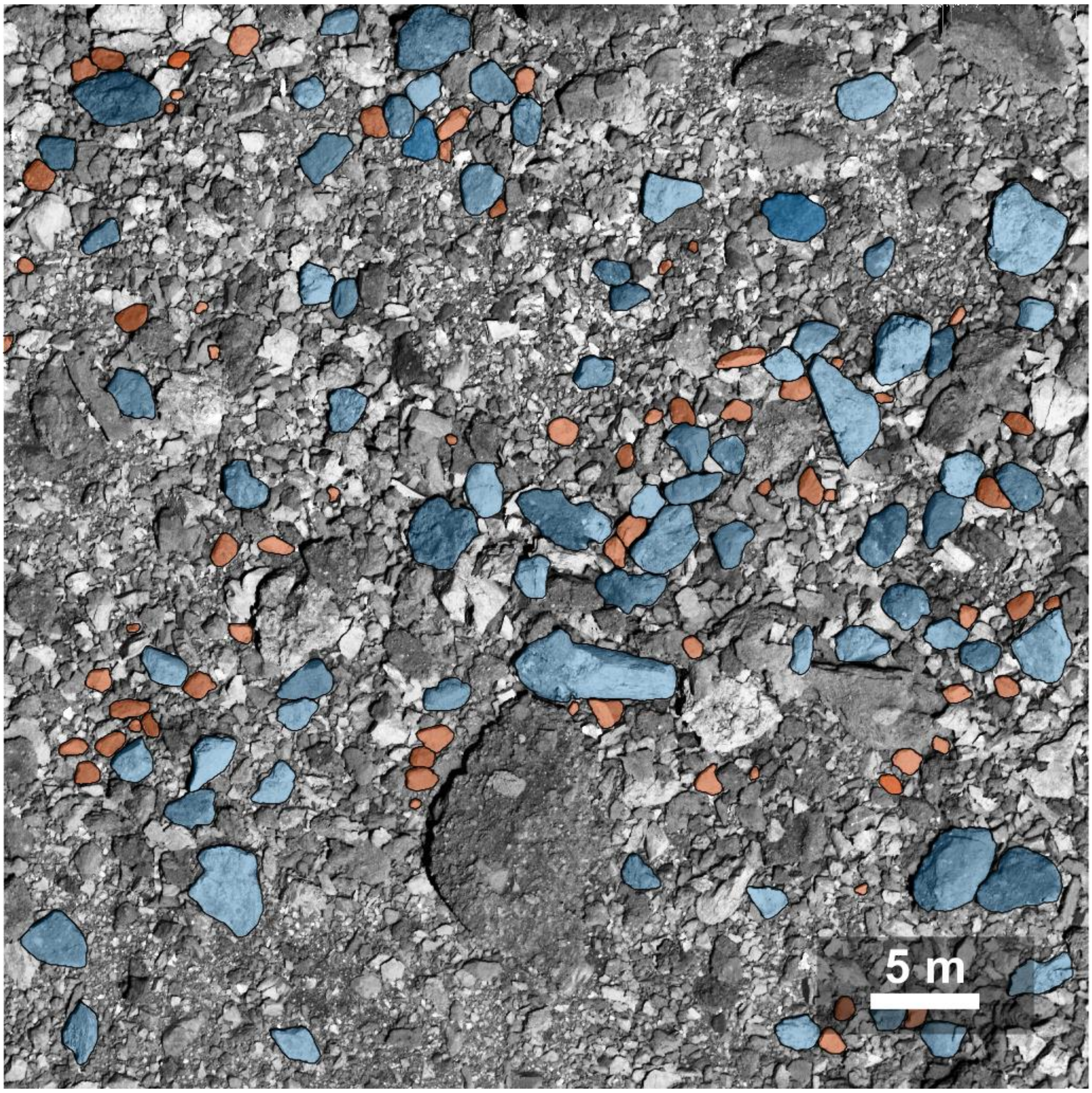

Supplementary Figure 14 | Bennu surface. Asteroid (101955) Bennu taken by OCAMS (20210407T033605S787_pol_iofL2pan). The image contrast has been enhanced with a CLAHE filtering for visualisation purposes only. Boulders selected and analysed are coloured in blue and red. The red boulders indicate the smaller resolved boulders (<30 px), which haven't been included in the analysis of the resolution dependant morphological parameters.

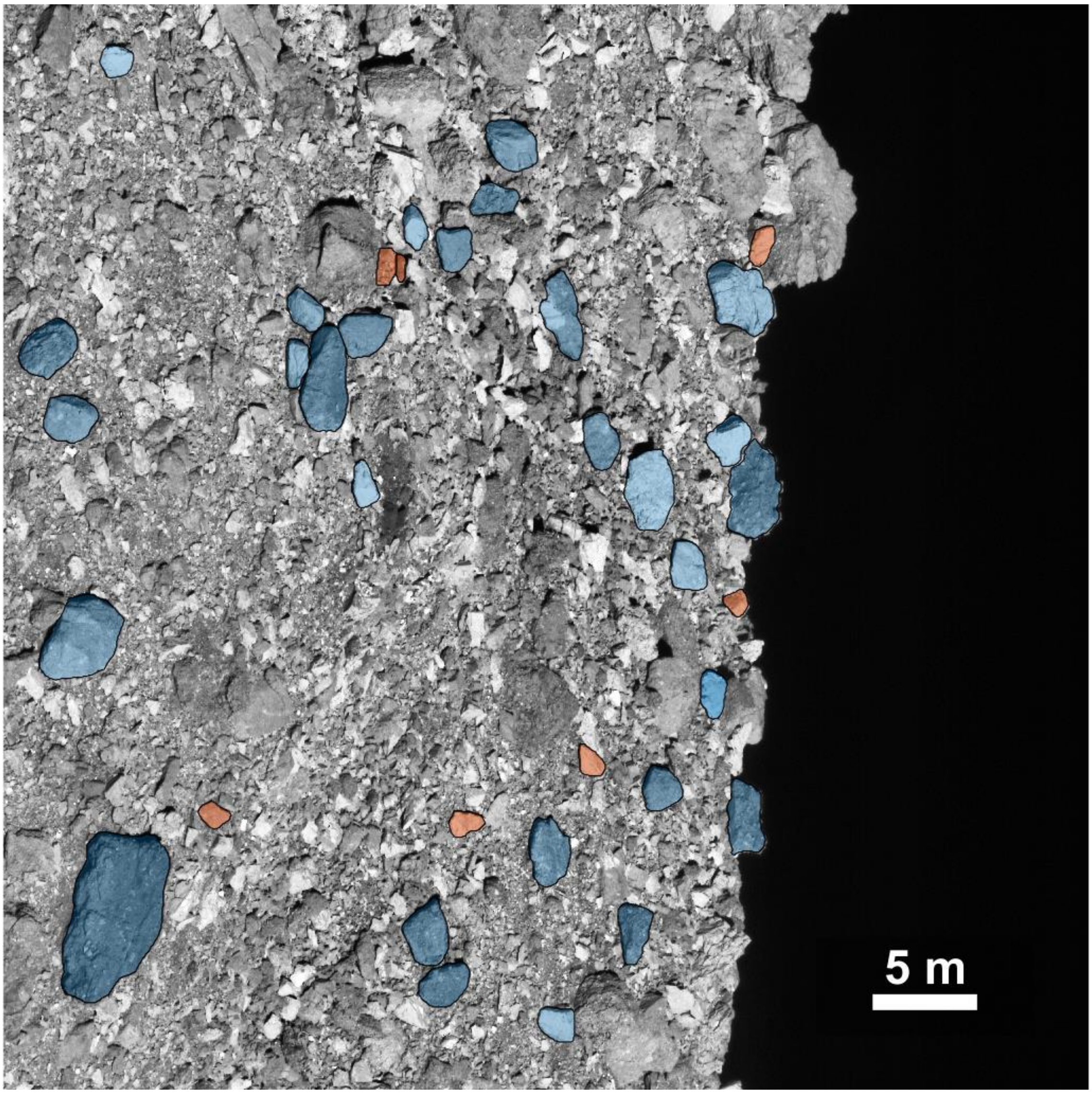

Supplementary Figure 15 | Bennu surface. Asteroid (101955) Bennu taken by OCAMS (20210407T044147S537_pol_iofL2pan). The image contrast has been enhanced with a CLAHE filtering for visualisation purposes only. Boulders selected and analysed are coloured in blue and red. The red boulders indicate the smaller resolved boulders (<30 px), which haven't been included in the analysis of the resolution dependant morphological parameters.

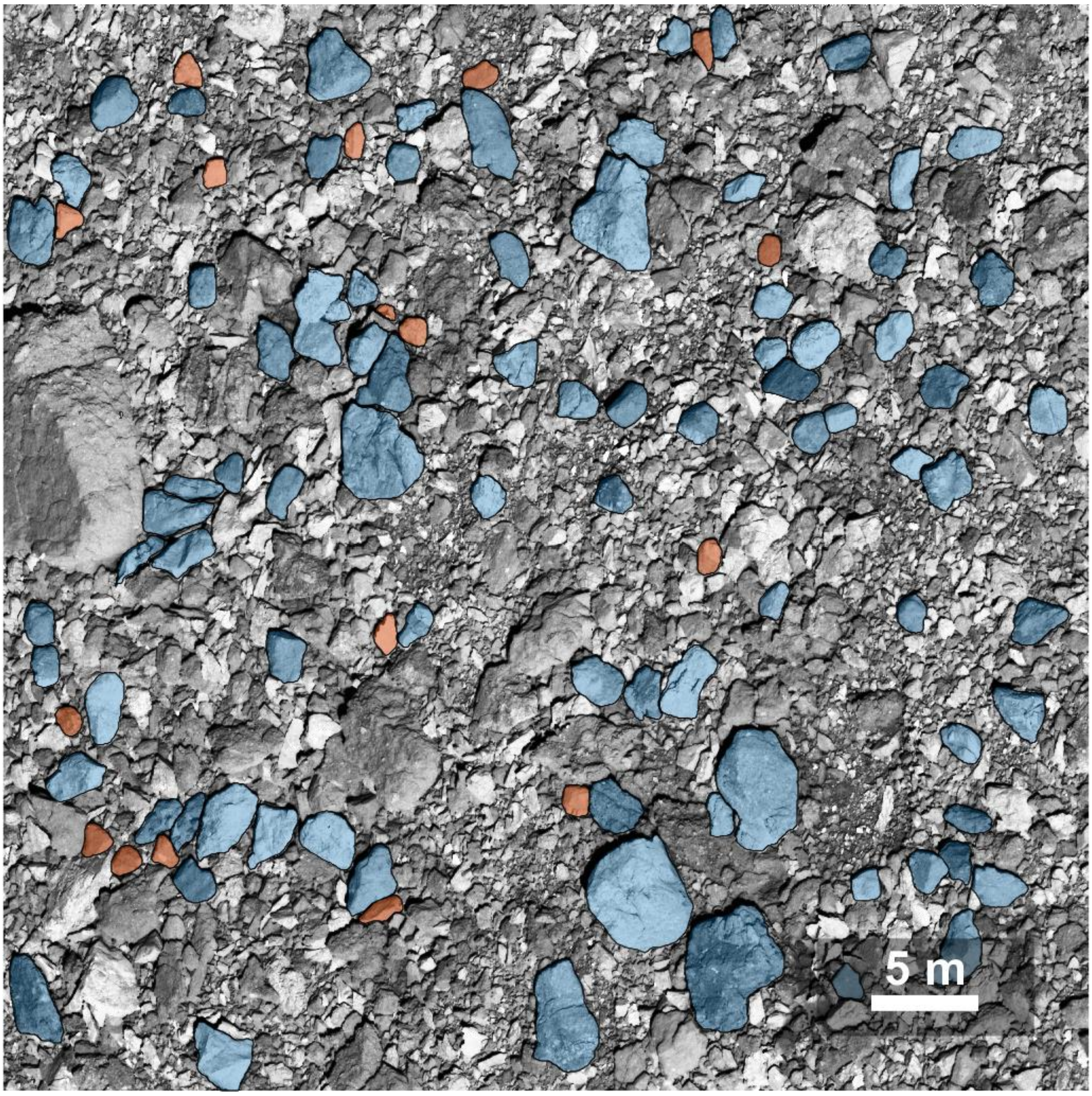

Supplementary Figure 16 | Bennu surface. Asteroid (101955) Bennu taken by OCAMS (20210407T044726S838_pol_iofL2pan). The image contrast has been enhanced with a CLAHE filtering for visualisation purposes only. Boulders selected and analysed are coloured in blue and red. The red boulders indicate the smaller resolved boulders (<30 px), which haven't been included in the analysis of the resolution dependant morphological parameters.

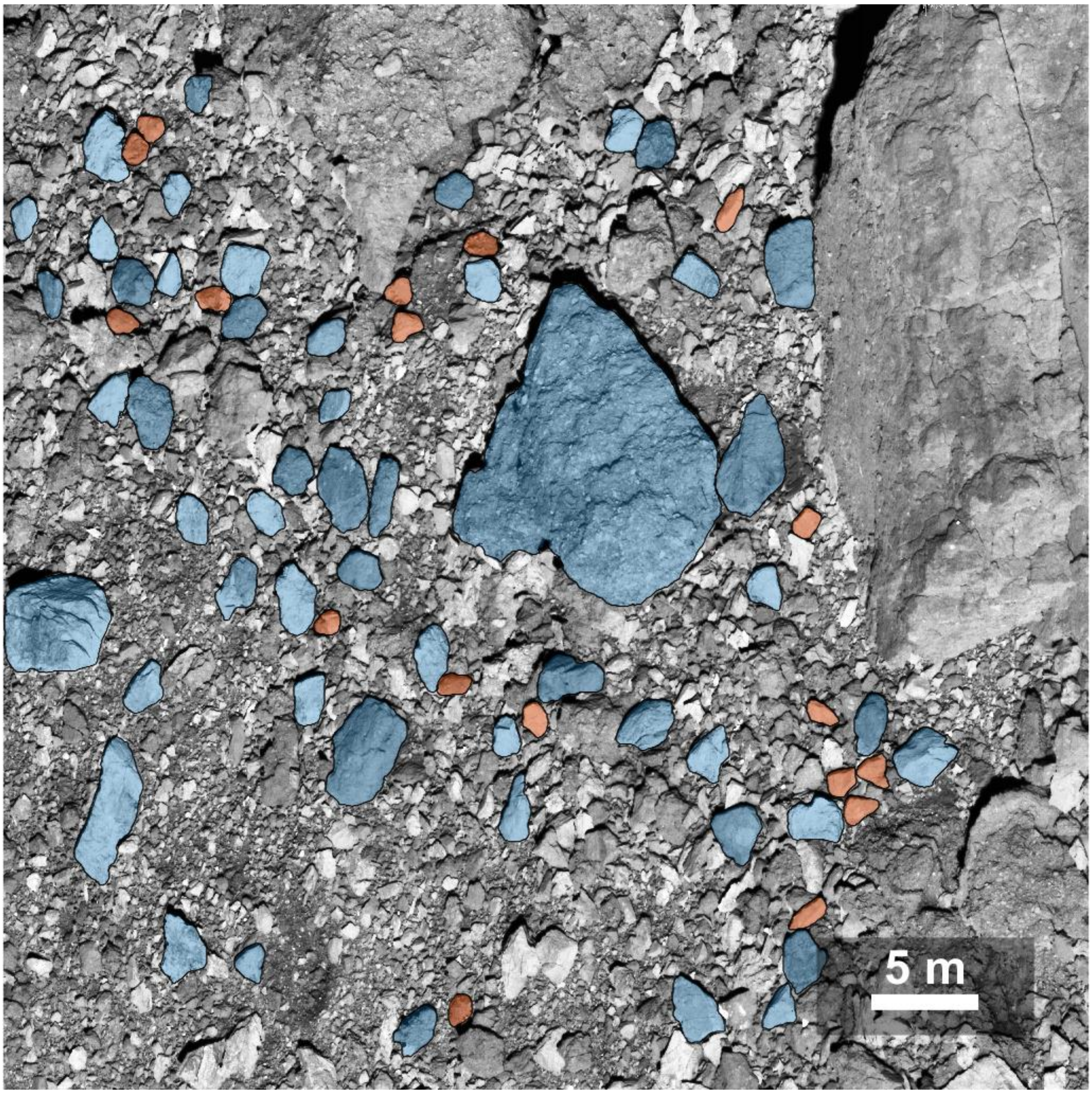

Supplementary Figure 17 | Bennu surface. Asteroid (101955) Bennu taken by OCAMS (20210407T053451S162_pol_iofL2pan). The image contrast has been enhanced with a CLAHE filtering for visualisation purposes only. Boulders selected and analysed are coloured in blue and red. The red boulders indicate the smaller resolved boulders (<30 px), which haven’t been included in the analysis of the resolution dependant morphological parameters.

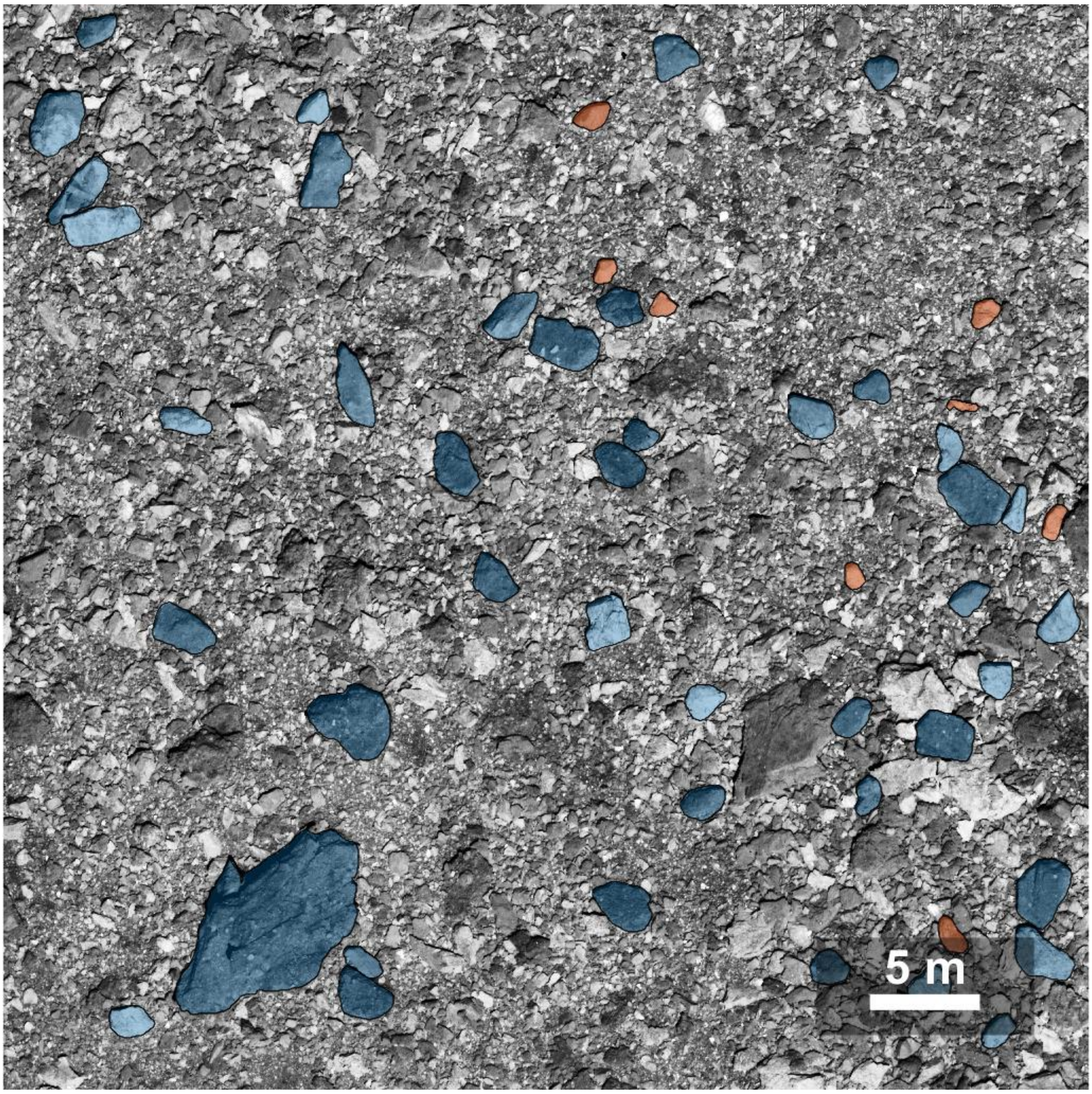

Supplementary Figure 18 | Bennu surface. Asteroid (101955) Bennu taken by OCAMS (20210407T054353S664_pol_iofL2pan). The image contrast has been enhanced with a CLAHE filtering for visualisation purposes only. Boulders selected and analysed are coloured in blue and red. The red boulders indicate the smaller resolved boulders (<30 px), which haven't been included in the analysis of the resolution dependant morphological parameters.

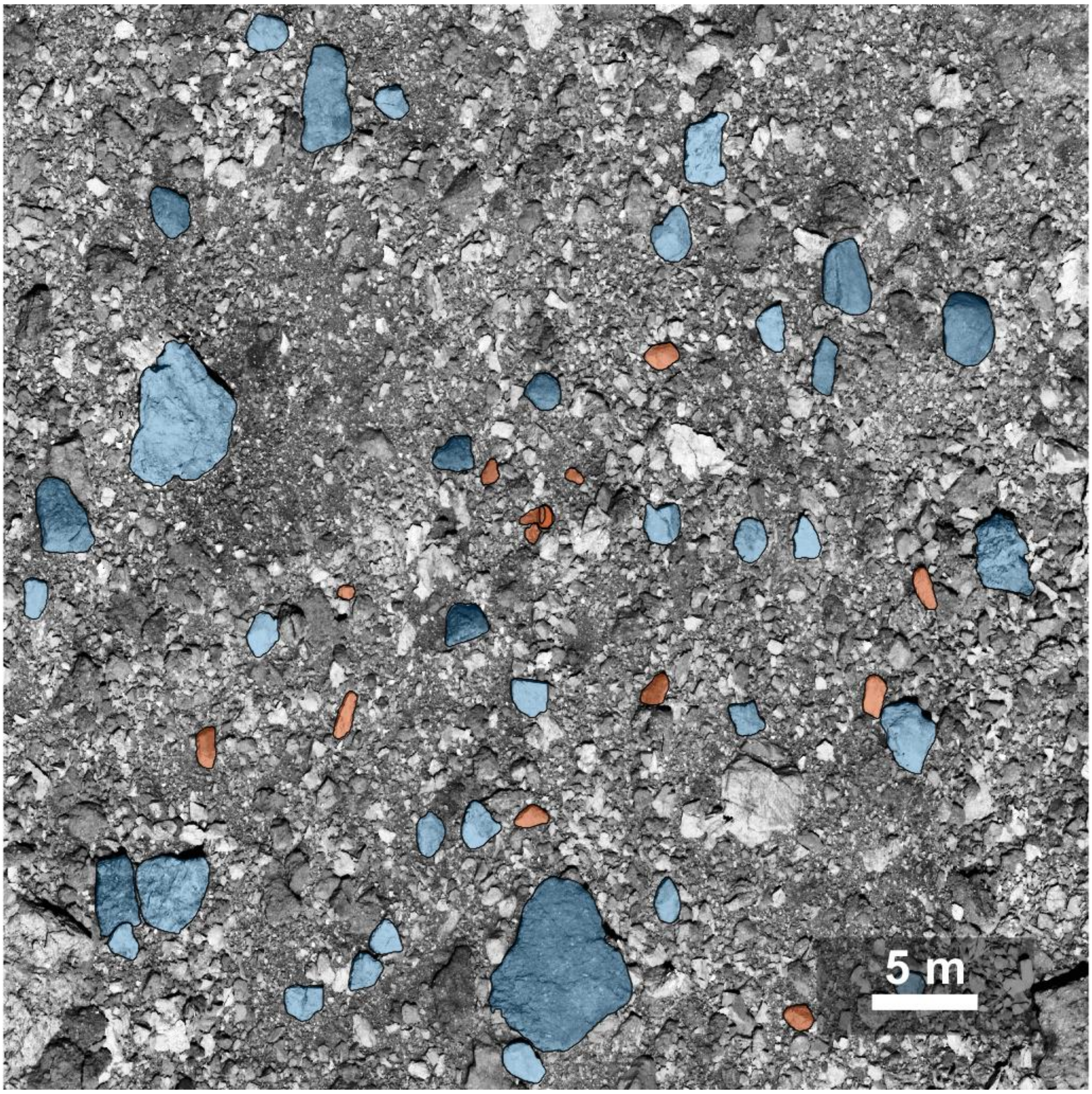

Supplementary Figure 19 | Bennu surface. Asteroid (101955) Bennu taken by OCAMS (20210407T060703S295_pol_iofL2pan). The image contrast has been enhanced with a CLAHE filtering for visualisation purposes only. Boulders selected and analysed are coloured in blue and red. The red boulders indicate the smaller resolved boulders (<30 px), which haven't been included in the analysis of the resolution dependant morphological parameters.

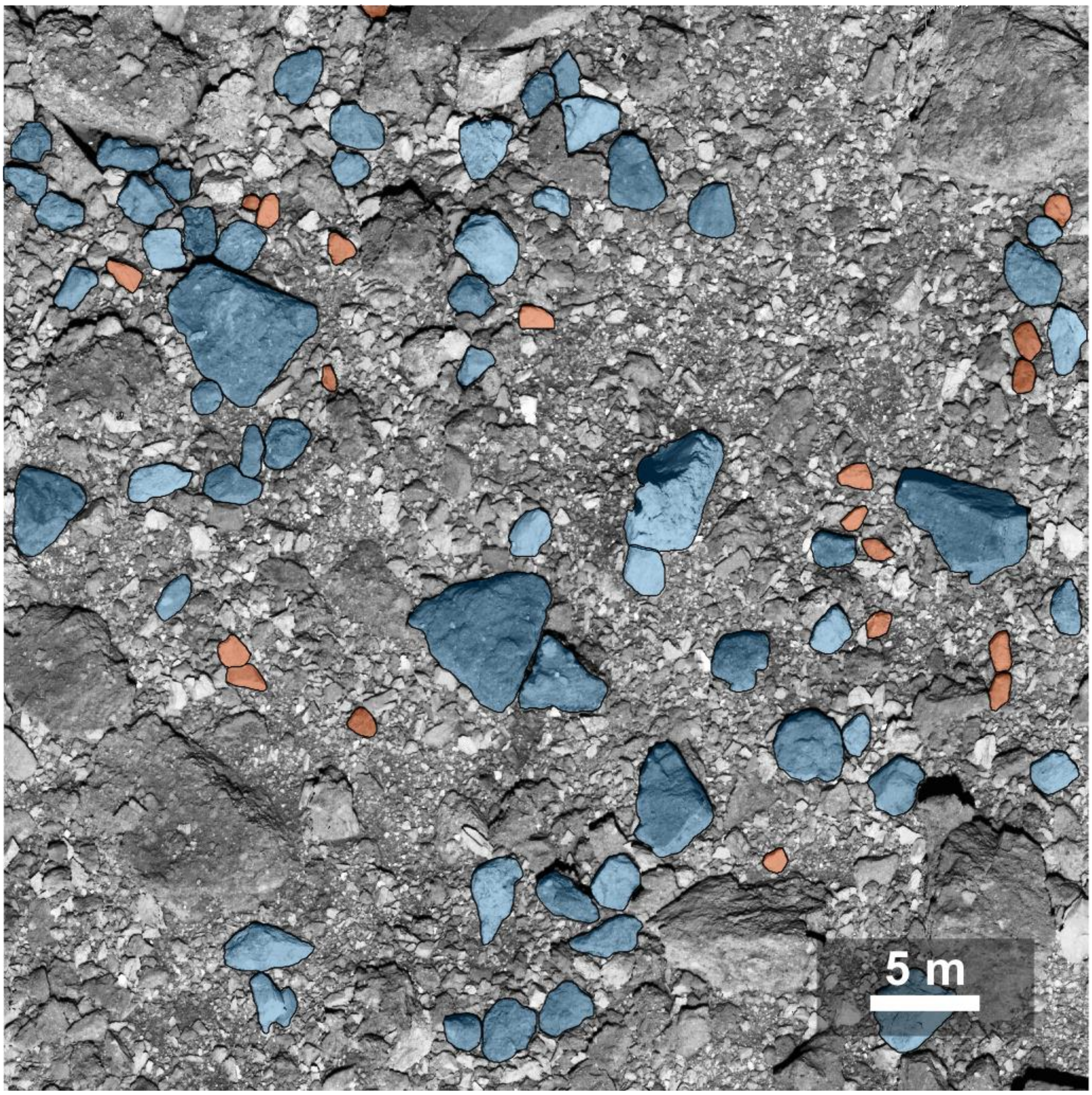

Supplementary Figure 20 | Bennu surface. Asteroid (101955) Bennu taken by OCAMS (20210407T062633S202_pol_iofL2pan). The image contrast has been enhanced with a CLAHE filtering for visualisation purposes only. Boulders selected and analysed are coloured in blue and red. The red boulders indicate the smaller resolved boulders (<30 px), which haven't been included in the analysis of the resolution dependant morphological parameters.

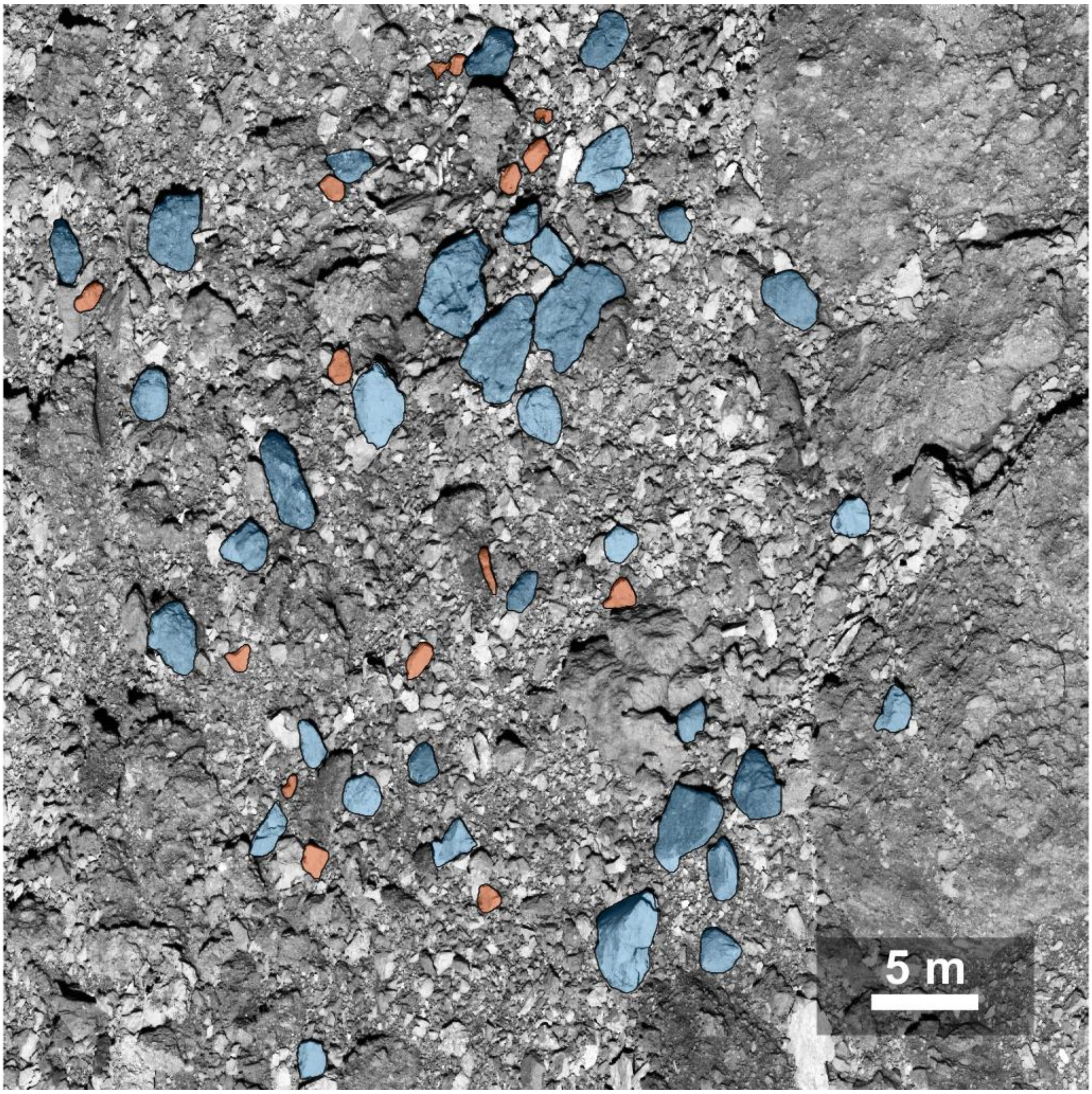

Supplementary Figure 21 | Bennu surface. Asteroid (101955) Bennu taken by OCAMS (20210407T063137S357_pol_iofL2pan). The image contrast has been enhanced with a CLAHE filtering for visualisation purposes only. Boulders selected and analysed are coloured in blue and red. The red boulders indicate the smaller resolved boulders (<30 px), which haven't been included in the analysis of the resolution dependant morphological parameters.

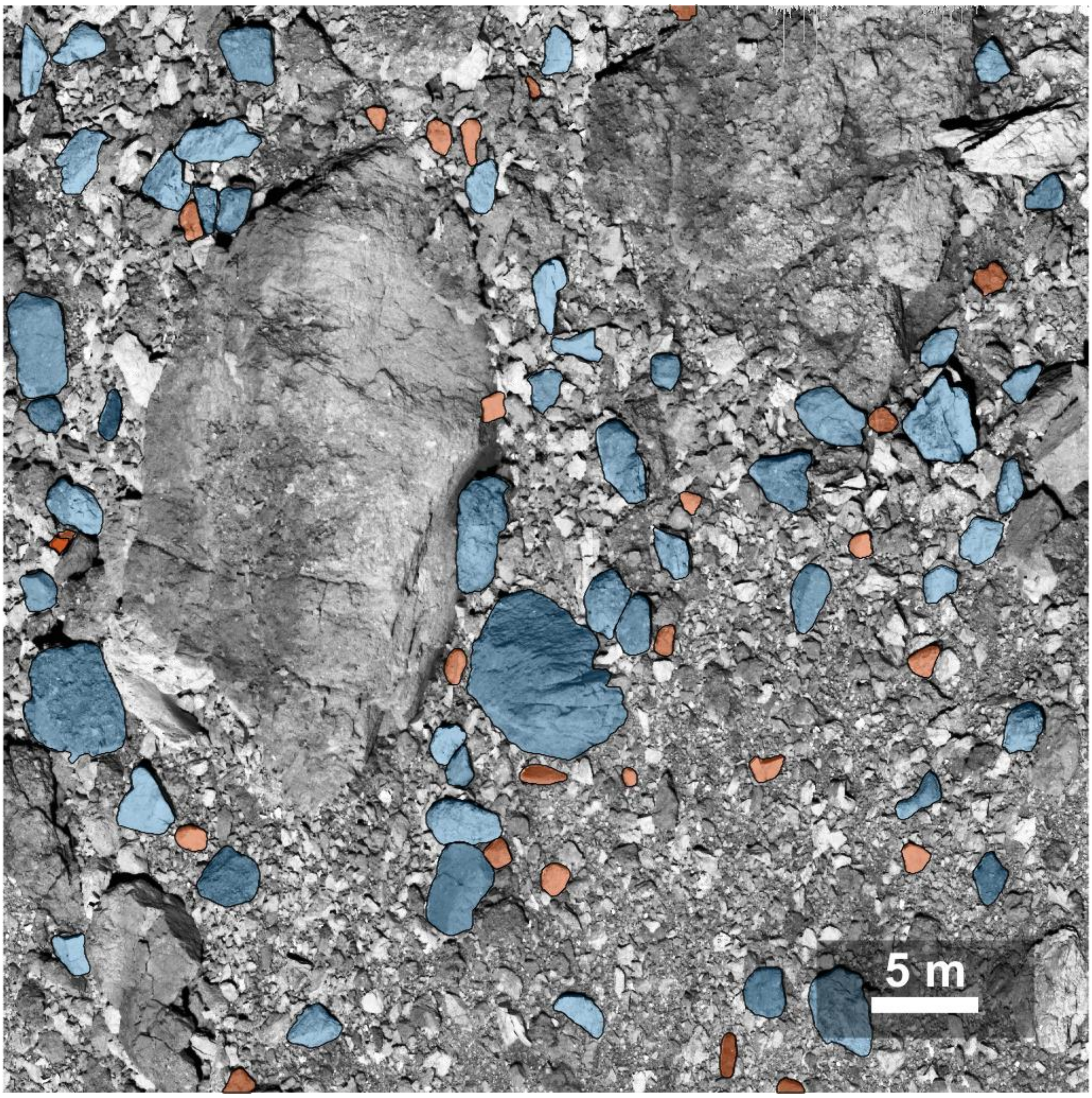

Supplementary Figure 22 | Bennu surface. Asteroid (101955) Bennu taken by OCAMS (20210407T070929S250_pol_iofL2pan). The image contrast has been enhanced with a CLAHE filtering for visualisation purposes only. Boulders selected and analysed are coloured in blue and red. The red boulders indicate the smaller resolved boulders (<30 px), which haven't been included in the analysis of the resolution dependant morphological parameters.

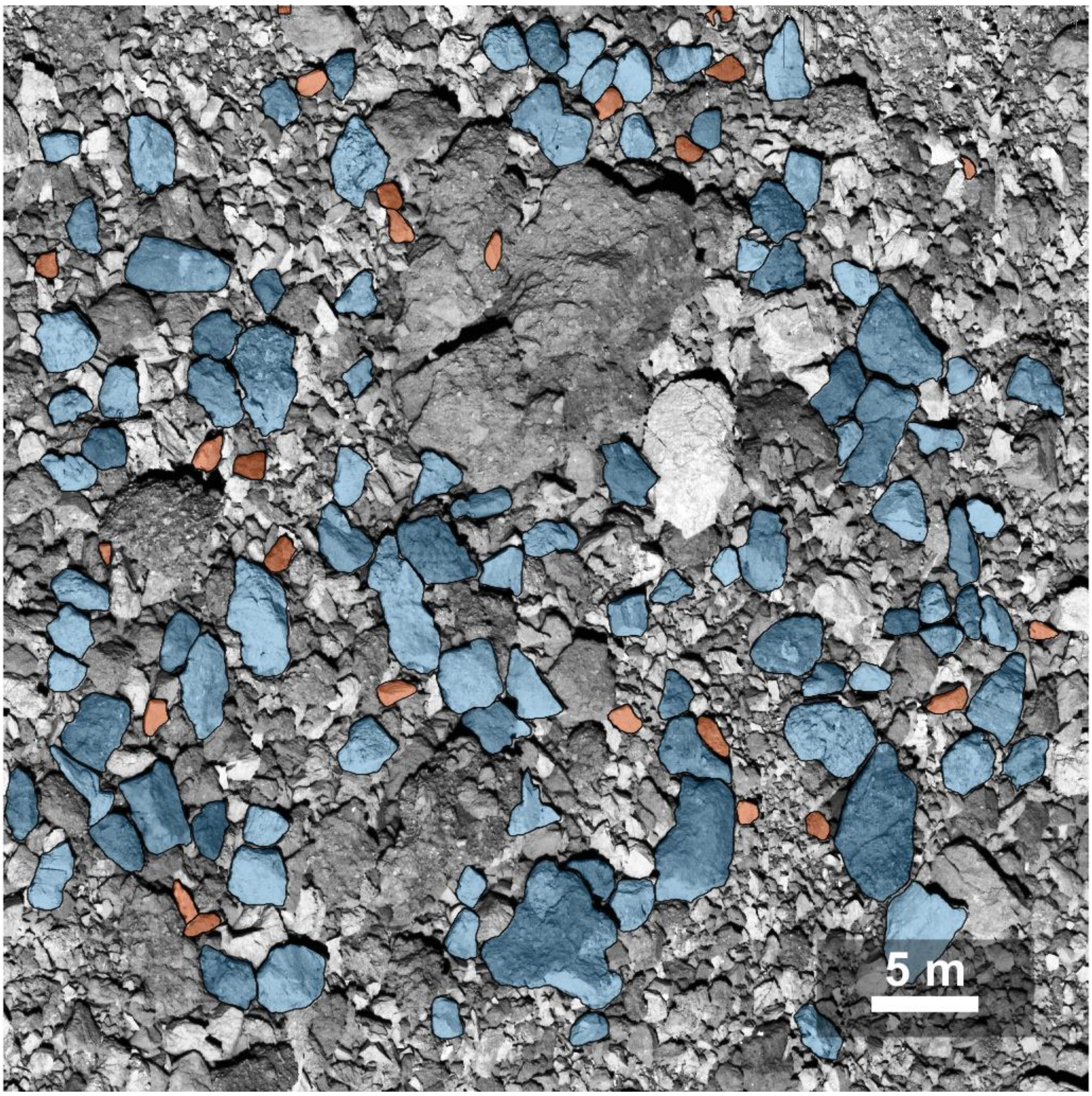

Supplementary Figure 23 | Bennu surface. Asteroid (101955) Bennu taken by OCAMS (20210407T072730S766_pol_iofL2pan). The image contrast has been enhanced with a CLAHE filtering for visualisation purposes only. Boulders selected and analysed are coloured in blue and red. The red boulders indicate the smaller resolved boulders (<30 px), which haven't been included in the analysis of the resolution dependant morphological parameters.

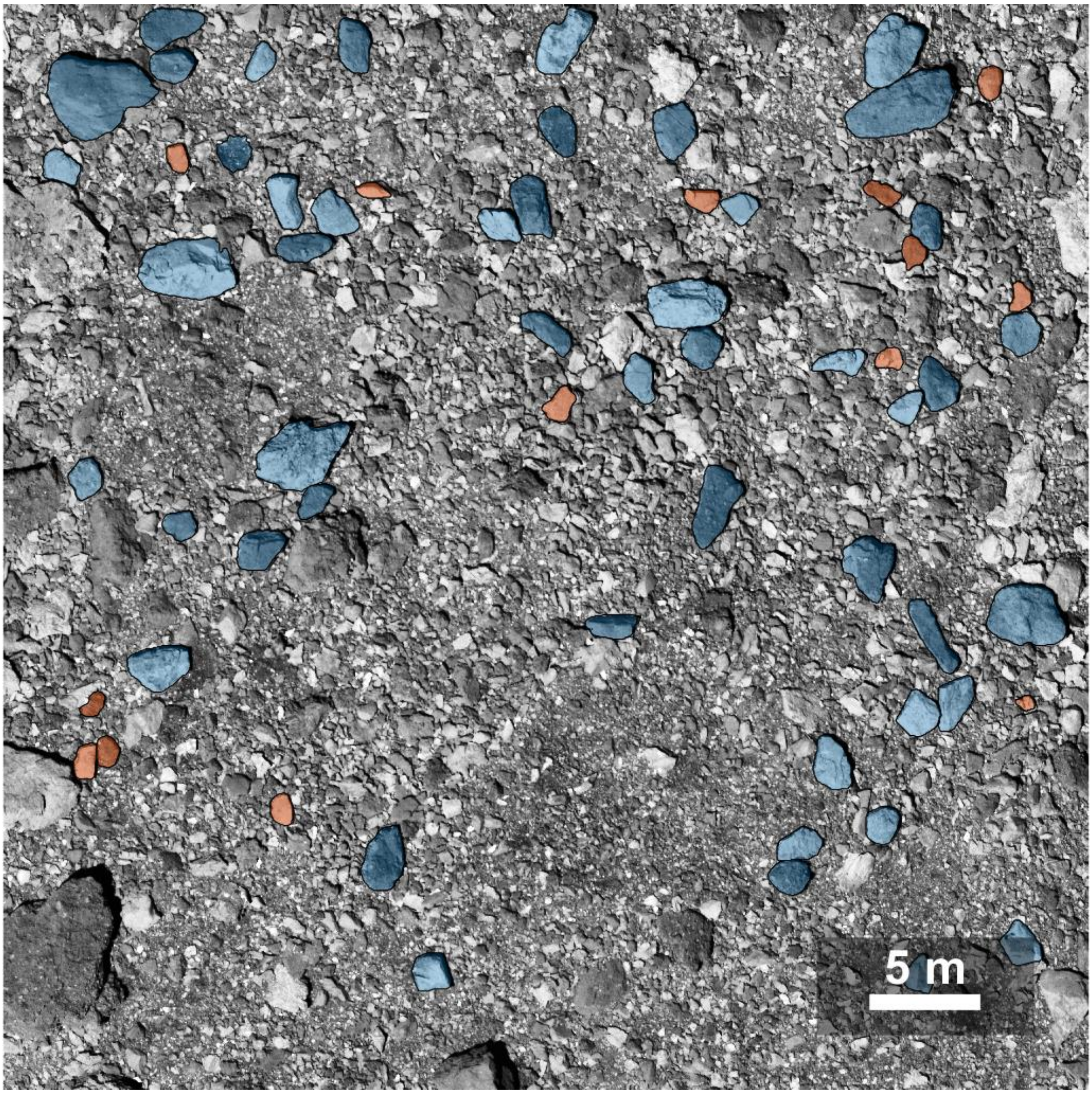

Supplementary Figure 24 | Bennu surface. Asteroid (101955) Bennu taken by OCAMS (20210407T092153S748_pol_iofL2pan). The image contrast has been enhanced with a CLAHE filtering for visualisation purposes only. Boulders selected and analysed are coloured in blue and red. The red boulders indicate the smaller resolved boulders (<30 px), which haven't been included in the analysis of the resolution dependant morphological parameters.

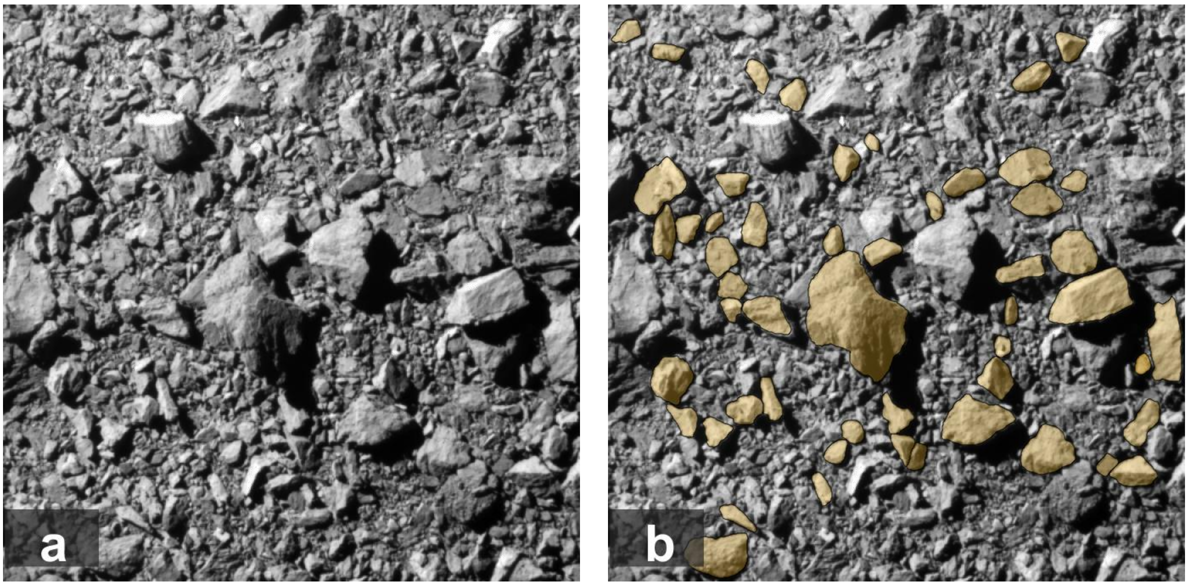

Supplementary Figure 25 | Last full image of Dimorphos surface before and after automatic segmentation. Dimorphos of the (65803) Dydimos | Dimorphos binary system taken by DRACO (*dart_0401930049_43695_02_iof*). a) the raw image used as input for the image segmentation. b) the raw image with the boulders (identified in yellow) detected after the two-step segmentation process.

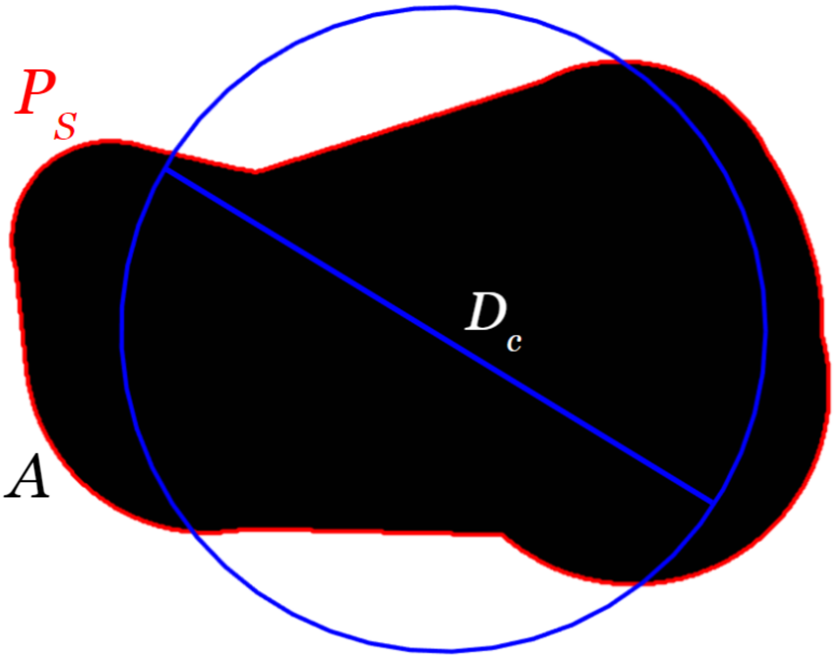

Supplementary Figure 26 | A particle and its equivalent diameter. A two-dimensional particle is shown alongside a blue circle with the same projected area (A) as the particle. This circle has a diameter D_c and perimeter P_S in red. This artificial particle shape will be used throughout this section to demonstrate the variables used in calculating morphological parameters.

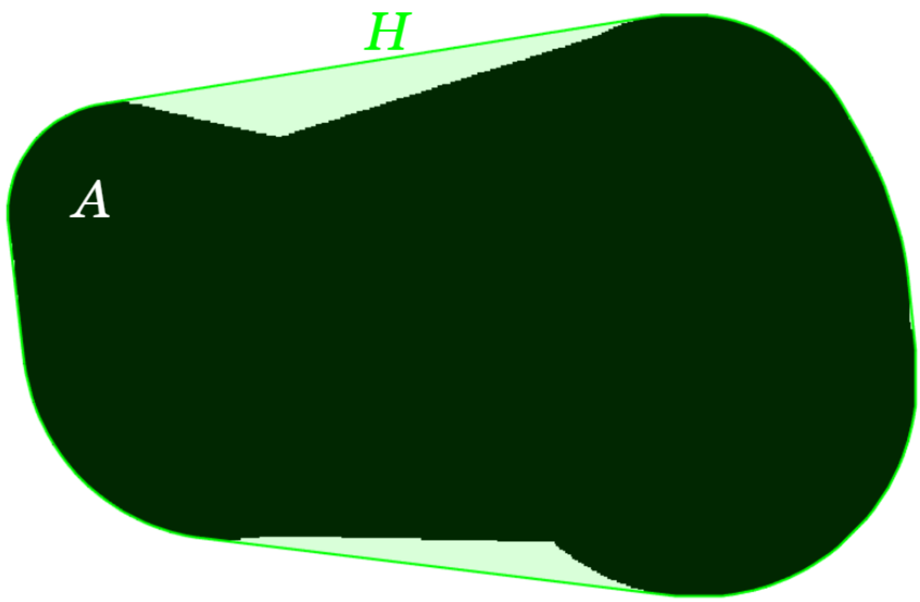

Supplementary Figure 27 | A particle and its convex hull. Convex hull in green of a two-dimensional particle. *H* is the area of the convex hull and *A* the area of the particle.

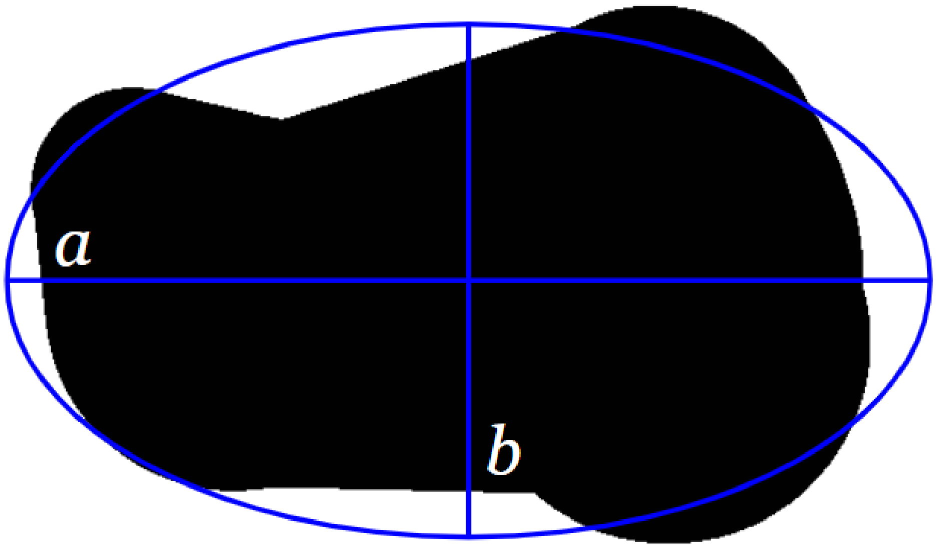

Supplementary Figure 28 | A particle and its fitted ellipse. Fitted ellipse of a particle and its axis, *a*, the major axis and *b*, the minor axis.

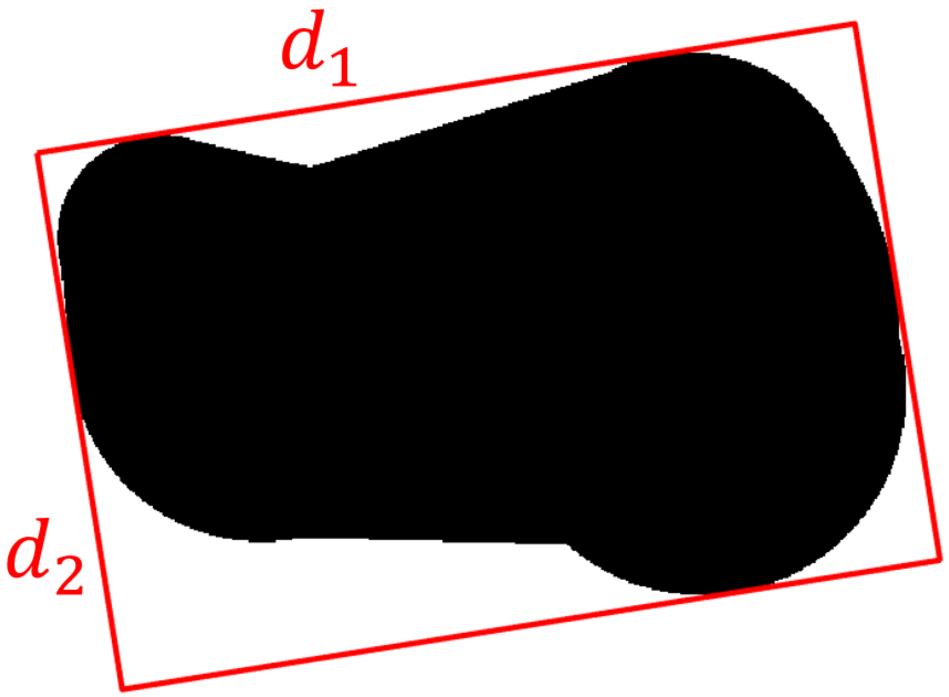

Supplementary Figure 29 | A particle and its bounding box. A particle and its associated minimal bounding box with d_1 and d_2, the box length and width, respectively.

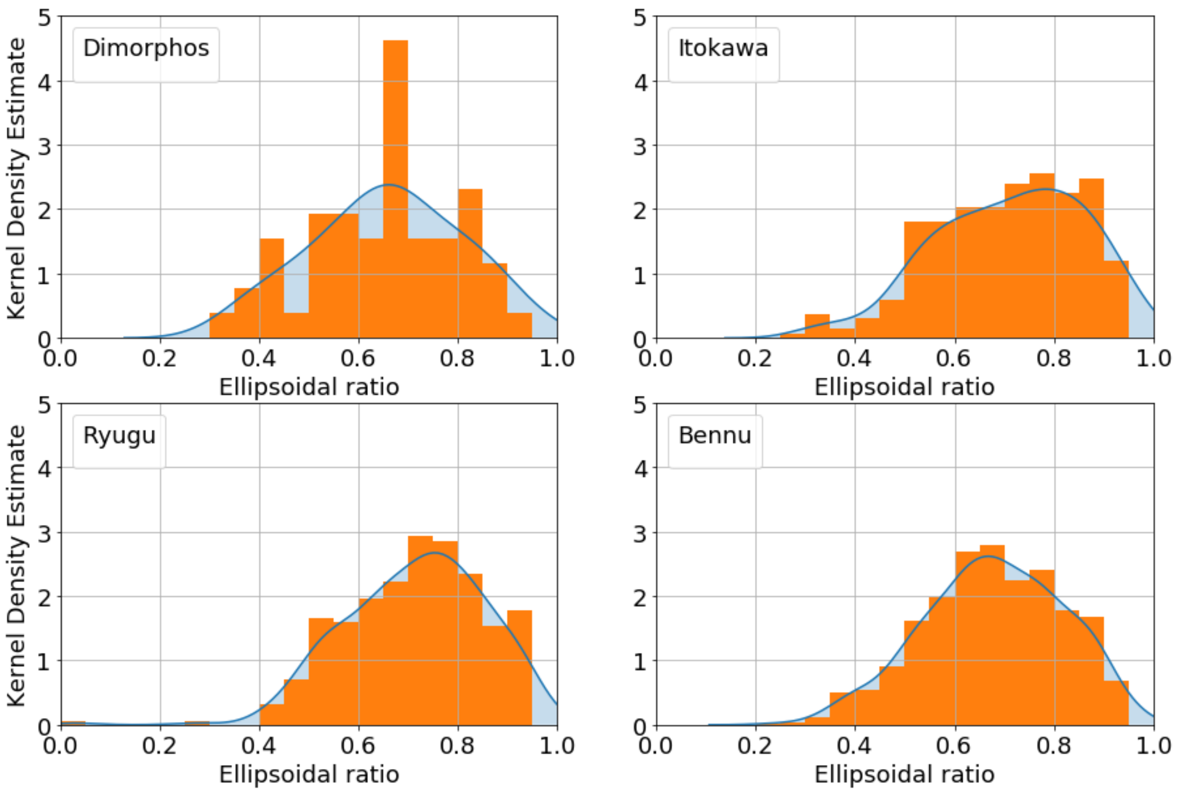

Supplementary Figure 30 | Distributions of the ellipsoidal ratios of surface boulders. The orange histogram shows the distribution of the apparent axial ratio of boulders on the surface and the blue shaded region represents the kernel density estimate of the apparent axial ratio of these boulders.

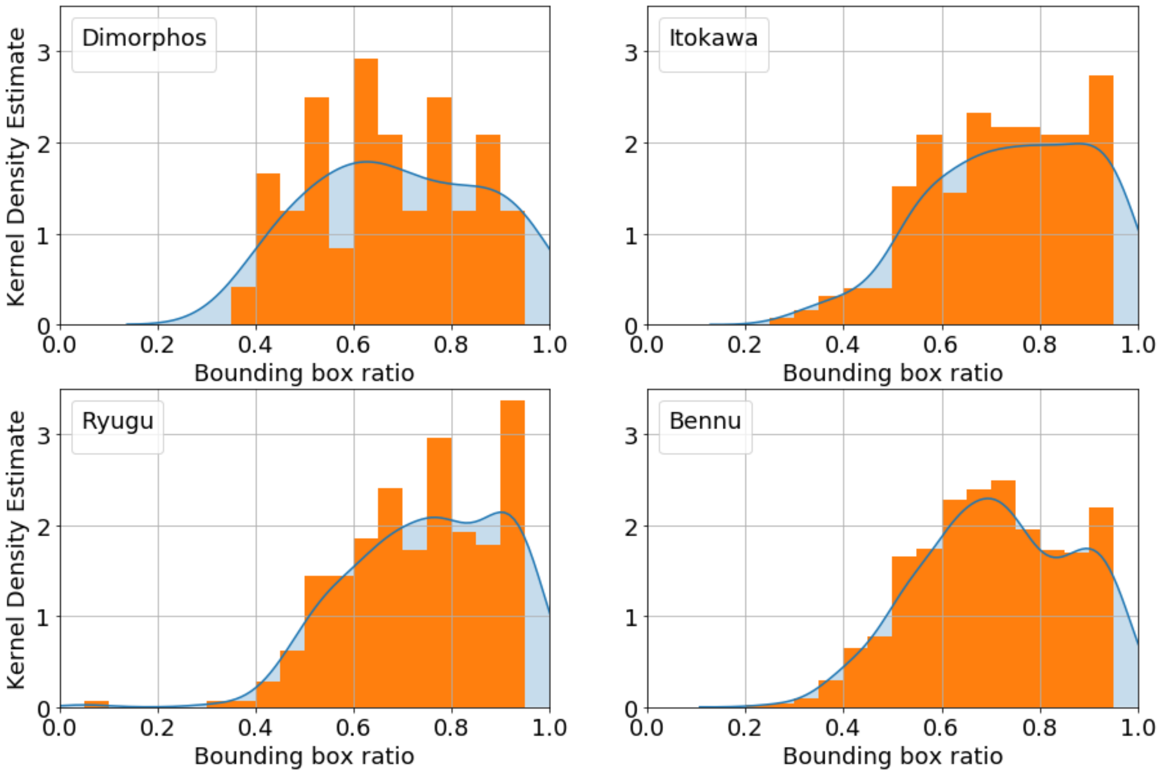

Supplementary Figure 31 | Distributions of the bounding box ratios of surface boulders. The orange histogram shows the distribution of the apparent axial ratio of boulders on the surface and the blue shaded region represents the kernel density estimate of the apparent axial ratio of these boulders.

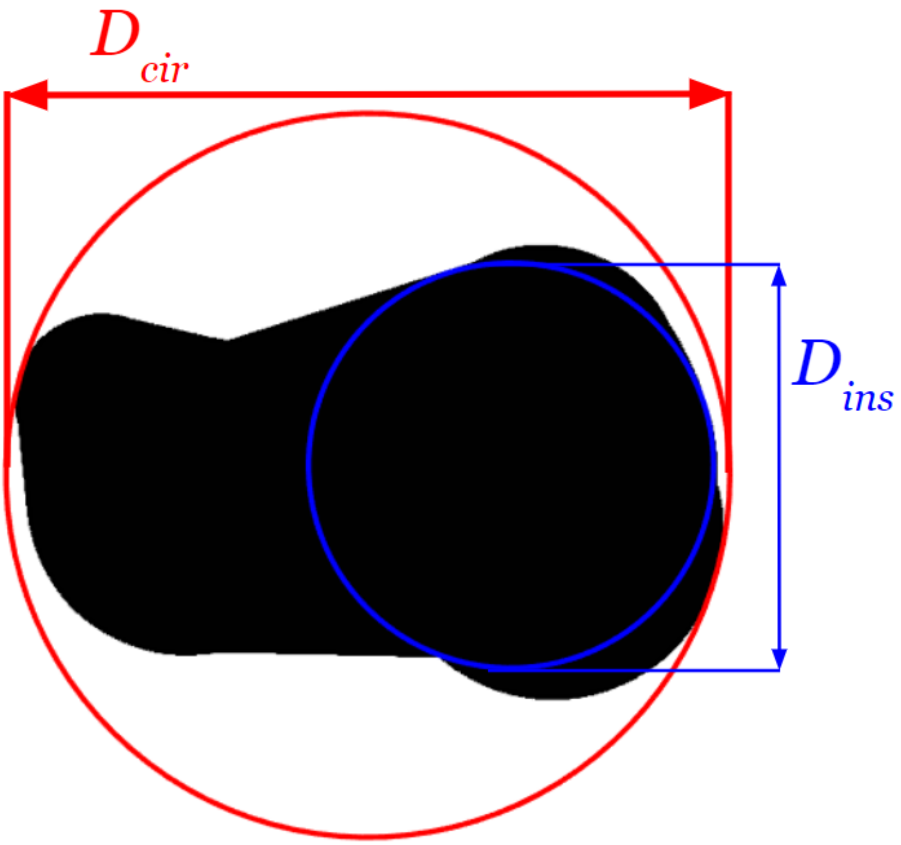

Supplementary Figure 32 | A particle, its maximum inscribed circle and its minimum circumscribed circle. The maximum inscribed circle of a particle has a diameter D_{ins} and the minimum circumscribed circle of a particle a diameter D_{cir}.

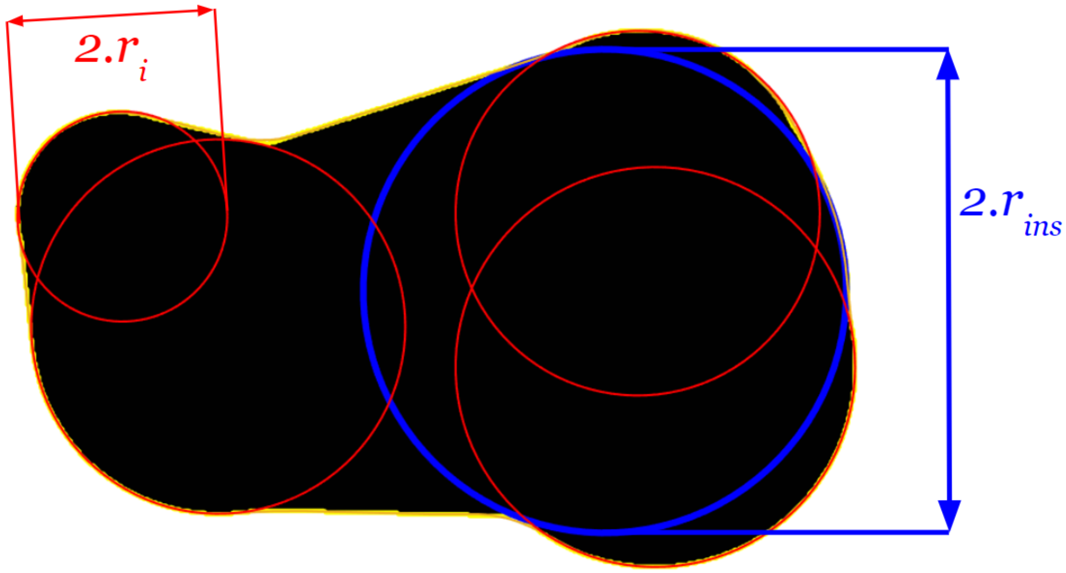

Supplementary Figure 33 | A particle, its maximum inscribed circle and its corner circles. The maximum inscribed circle has a radius of r_{ins} and the corner circles has radii of r_i.

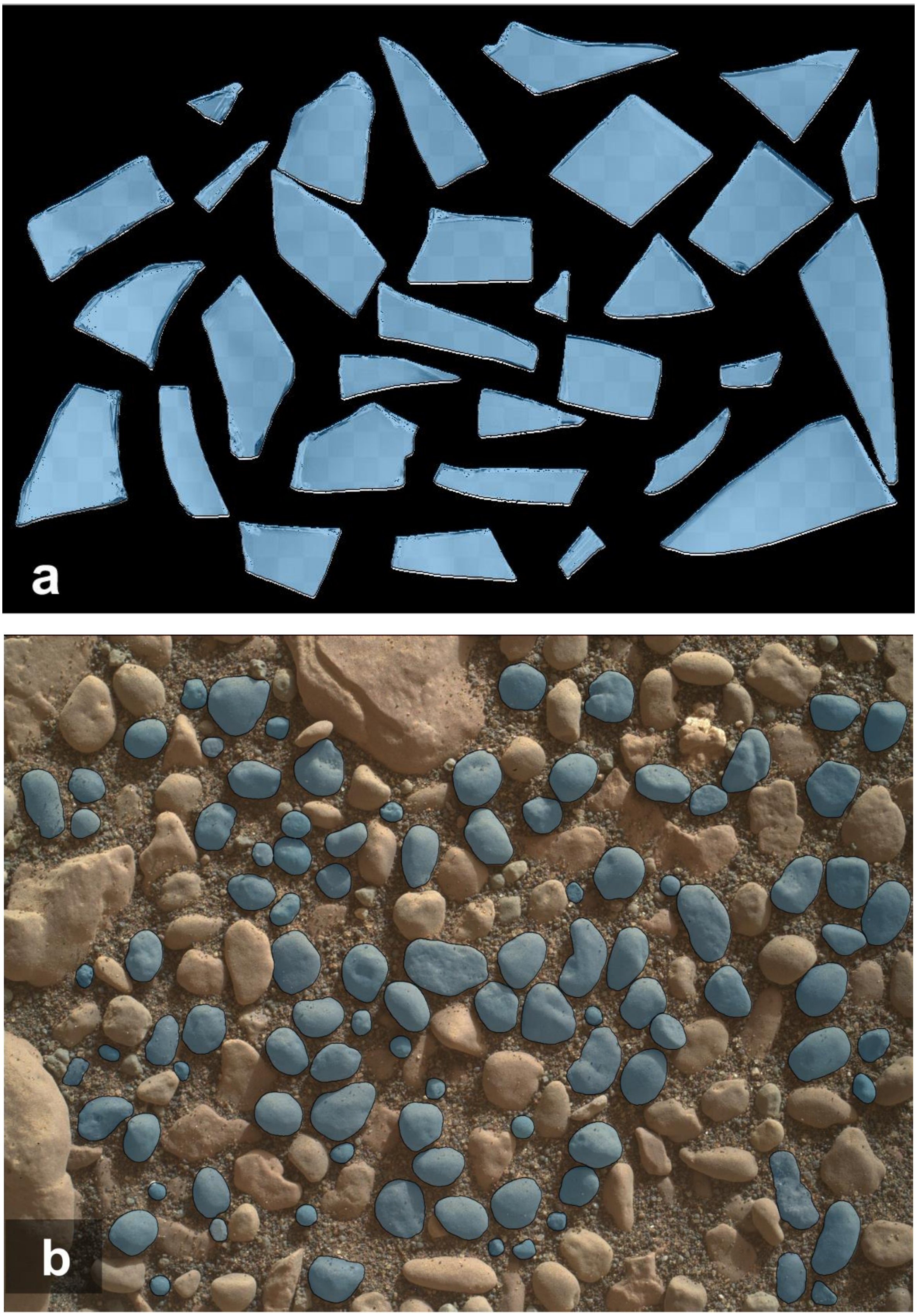

Supplementary Figure 34 | Images used to compute extreme roundness values. a) Image of broken glass representing an angular sample. b) Image of rounded pebbles on the surface of Mars taken by MAHLI on sol 2356 of the Mars Science Laboratory Mission. The particles coloured in blue indicates the shapes analysed for the roundness computation.

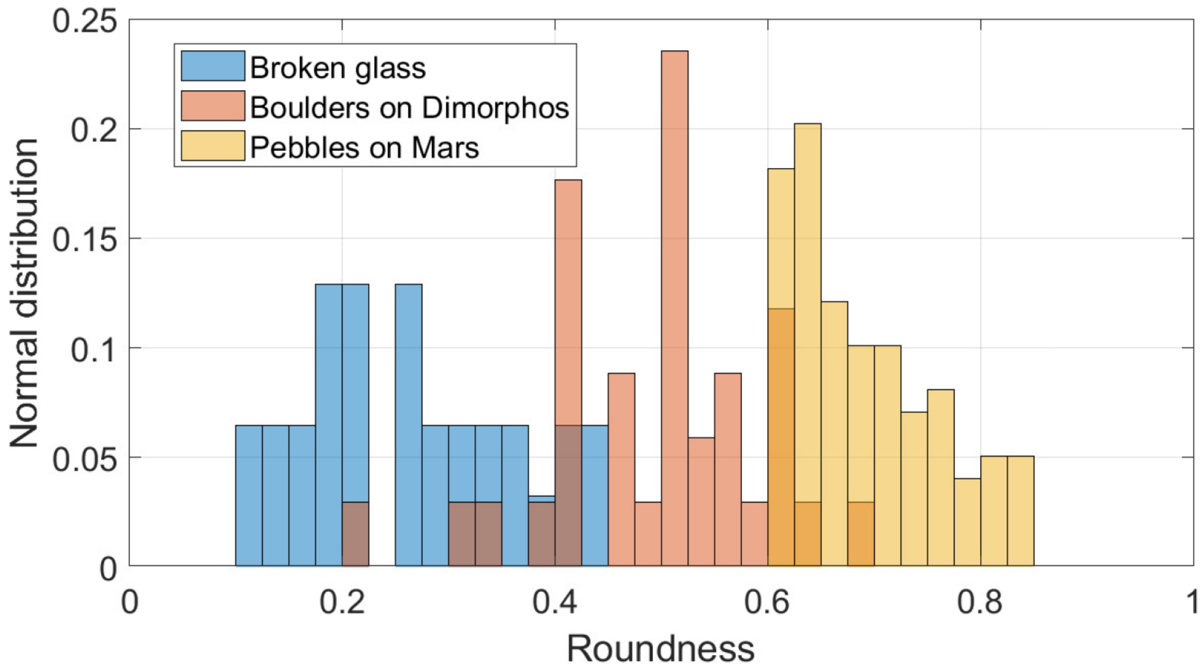

Supplementary Figure 35 | Distributions of the roundness for broken glass, Dimorphos and Mars pebbles. Normal distribution of the roundness computed for three different samples: broken glass (in blue), boulders on Dimorphos (orange), and pebbles on Mars (in yellow).

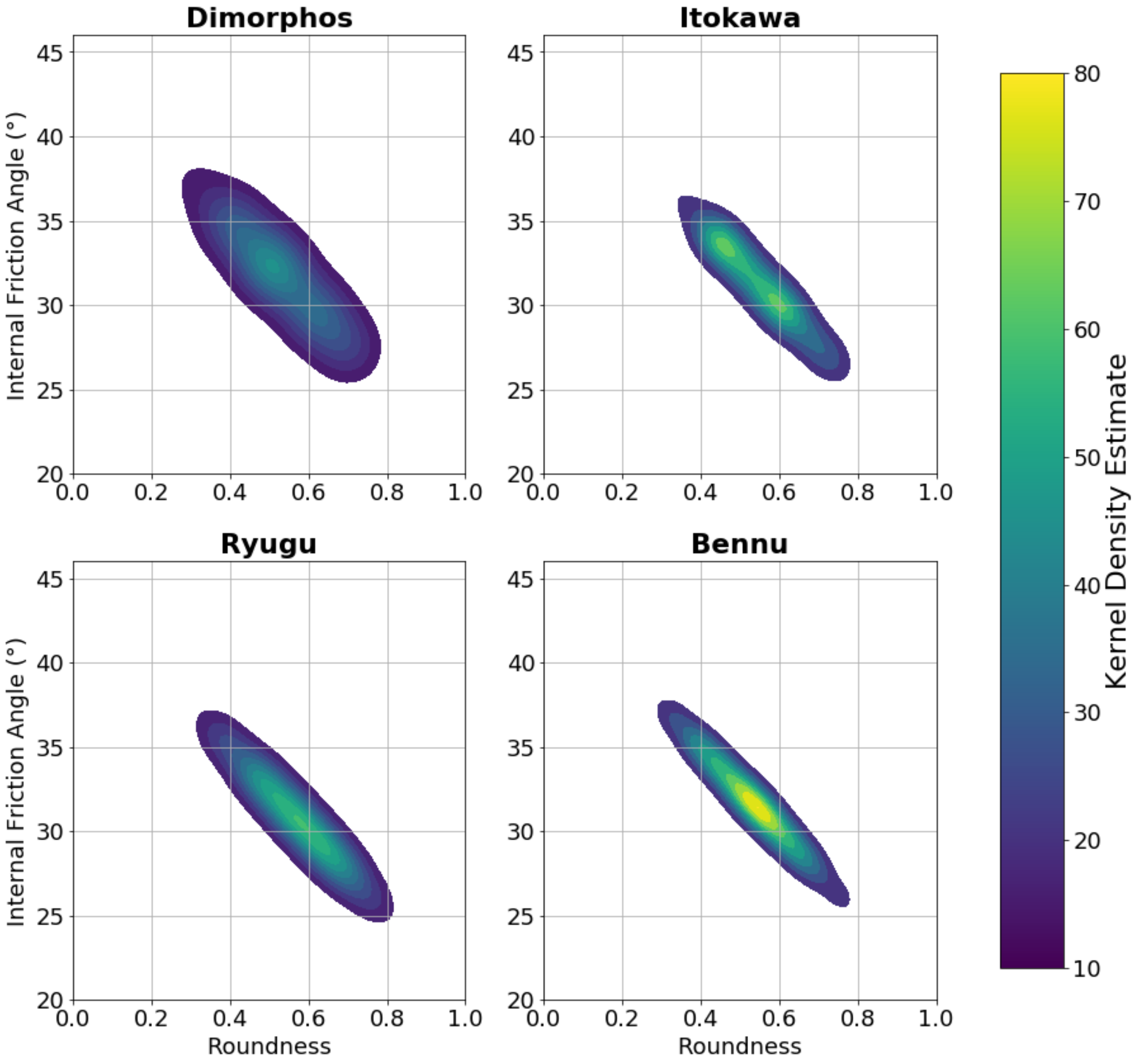

Supplementary Figure 36 | Empirical relationships between internal friction and roundness. Bivariate kernel density estimates between internal friction angle and roundness for Dimorphos, Itokawa, Ryugu and Bennu.

Supplementary Tables

Body	File name	Resolution (m/px)	Phase Angle (°)	Emission Angle (°)	Latitude	Longitude
Dimorphos	dart_0401930049_43695_02_iof	0.055	59.73	0.58-74.59	-9.3	262.6
Itokawa	st_2530297837_v	0.065	17.93	36.62	-4.5	179.1
Itokawa	st_2532629277_v	0.075	10.66	51.5	-20.9	84.4
Itokawa	st_2539429953_v	0.022	16.71	47.82	-6.1	89.4
Itokawa	st_2539437177_v	0.058	17.44	47.24	9.7	53.5
Itokawa	st_2539444467_v	0.062	16.05	24.24	9.1	49.8
Itokawa	st_2539451609_v	0.065	15.29	19.28	10.5	50.4
Itokawa	st_2539467169_v	0.050	16.56	68.78	9.8	153.2
Ryugu	hyb2_onc_20180921_034938_tvf	0.063	-	-	11.3	141.8
Ryugu	hyb2_onc_20180921_040634_tvf	0.055	-	-	14.7	127.5
Ryugu	hyb2_onc_20180921_041826_tvf	0.081	-	-	13.8	115.5
Ryugu	hyb2_onc_20181003_003121_tnf	0.095	-	-	-12.6	27.1
Ryugu	hyb2_onc_20181003_021156_tvf	0.076	-	-	-23.7	312.3
Ryugu	hyb2_onc_20181015_130841_tuf	0.074	-	-	4.3	235.4
Ryugu	hyb2_onc_20181015_133137_tpf	0.060	-	-	5.1	217.6
Ryugu	hyb2_onc_20190710_232532_tvf	0.096	-	-	-0.1	5.4
Bennu	20210407T070929S250_pol_iofL2pan	0.050	10.43	65.93	-59.9	345.2
Bennu	20210407T033629S004_pol_iofL2pan	0.050	8.42	48.4	-50.5	285.6
Bennu	20210407T044726S838_pol_iofL2pan	0.049	7.98	51.21	-47.3	188.1
Bennu	20210407T053451S162_pol_iofL2pan	0.049	8.30	63.85	-61.9	124.5
Bennu	20210407T063137S357_pol_iofL2pan	0.050	9.41	63.27	-59.4	42.0
Bennu	20210407T062633S202_pol_iofL2pan	0.048	8.89	15.92	-13.7	45.9
Bennu	20210407T054353S664_pol_iofL2pan	0.048	8.60	5.30	-6.7	104.9
Bennu	20210407T033605S787_pol_iofL2pan	0.049	10.05	4.61	-4.0	282.1
Bennu	20210407T092153S748_pol_iofL2pan	0.048	12.61	4.51	-2.3	161.4
Bennu	20210407T060703S295_pol_iofL2pan	0.050	9.58	69.96	60.3	60.6
Bennu	20210407T044147S537_pol_iofL2pan	0.051	11.07	88.02	76.0	161.2
Bennu	20210407T072730S766_pol_iofL2pan	0.050	9.29	64.61	57.8	317.8

Supplementary Table 1 | Observation data of the studied asteroids images. Available observation data used for image selection on Dimorphos, Itokawa, Ryugu and Bennu from PDS. Some information is not yet publicly available for Ryugu.
